# Understanding the complex network of objectively assessed cognition and self-reported psychological symptoms in people with multiple sclerosis

**DOI:** 10.1177/13524585241302173

**Published:** 2024-12-09

**Authors:** Maureen van Dam, Jantine G Röttgering, Ilse M Nauta, Brigit A de Jong, Martin Klein, Menno M Schoonheim, Bernard MJ Uitdehaag, Hanneke E Hulst, Linda Douw

**Affiliations:** MS Center Amsterdam, Department of Anatomy & Neurosciences, Vrije Universiteit Amsterdam, Amsterdam Neuroscience, Amsterdam UMC Location VUmc, Amsterdam, The Netherlands; Health, Medical and Neuropsychology Unit, Institute of Psychology, Leiden University, Leiden, The Netherlands; Medical Psychology, Vrije Universiteit Amsterdam, Amsterdam UMC Location, Amsterdam, The Netherlands; Brain Tumor Center, Cancer Center Amsterdam, Amsterdam, The Netherlands; MS Center Amsterdam, Neurology, Vrije Universiteit Amsterdam, Amsterdam Neuroscience, Amsterdam UMC Location VUmc, Amsterdam, The Netherlands; MS Center Amsterdam, Neurology, Vrije Universiteit Amsterdam, Amsterdam Neuroscience, Amsterdam UMC Location VUmc, Amsterdam, The Netherlands; Medical Psychology, Vrije Universiteit Amsterdam, Amsterdam UMC Location, Amsterdam, The Netherlands; Brain Tumor Center, Cancer Center Amsterdam, Amsterdam, The Netherlands; MS Center Amsterdam, Department of Anatomy & Neurosciences, Vrije Universiteit Amsterdam, Amsterdam Neuroscience, Amsterdam UMC Location VUmc, Amsterdam, The Netherlands; MS Center Amsterdam, Neurology, Vrije Universiteit Amsterdam, Amsterdam Neuroscience, Amsterdam UMC Location VUmc, Amsterdam, The Netherlands; Health, Medical and Neuropsychology Unit, Institute of Psychology, Leiden University, Leiden, The Netherlands; Leiden Institute for Brain and Cognition, Leiden, The Netherlands; MS Center Amsterdam, Department of Anatomy & Neurosciences, Vrije Universiteit Amsterdam, Amsterdam Neuroscience, Amsterdam UMC Location VUmc, Amsterdam, The Netherlands

**Keywords:** Multiple sclerosis, cognition, symptom network analysis, patient reported outcome measures, information processing speed

## Abstract

**Background:**

Literature on the intricate relationship between self-reported and objectively assessed cognitive functioning suggests a discrepancy between self-reported cognitive complaints (SCC) and actual test performance.

**Objectives:**

To investigate the interplay between patient-reported outcome measures (PROMs) and objective cognitive functioning using network analysis in people with multiple sclerosis (PwMS).

**Methods:**

We collected PROMs on anxiety, depression, fatigue and SCC, and cognitive functioning across six domains (*n* = 703 PwMS; 71% female, mean age = 46.3 ± 11.2 years). We constructed cognitive symptom networks using Gaussian Graphical Models, in which the aforementioned variables were presented as nodes linked by regularized partial correlations. We compared global network strength between relevant subgroups.

**Results:**

The networks primarily showed clustering of PROMs and cognitive domains into two separate modules, with weaker links connecting both modules. Global network strength was lower for PwMS with impaired information processing speed (IPS; indicating lower symptom interrelatedness) compared to those with preserved IPS (3.57 versus 4.51, *p* = 0.001), but not when comparing SCC subgroups (*p* = 0.140).

**Conclusions:**

Cognitive symptom networks deepen our understanding of the discrepancy between self-reported and objectively assessed cognitive functioning. Lower symptom interrelatedness in PwMS with impaired IPS might suggest a nonlinear relation between PROMs and cognitive domains, which depends on the cognitive status.

## Introduction

Cognitive impairment affects up to 65% of people with multiple sclerosis (PwMS), substantially impacting quality of life.^1^ Slowed information processing speed (IPS) is highly prevalent and among the first cognitive impairments in PwMS,^1^ possibly underlying other higher-level cognitive processes.^2^ Cognitive impairment is assessed using patient-reported outcome measures (PROMs) and formal neuropsychological testing,^1^ but prior research underlines a discordance between these methods, known as the subjective–objective discrepancy.^3–5^ Psychological factors, such as fatigue and depression, which are more common in MS than in the general population,^6,7^ may explain the discrepancy between self-reported cognitive complaints (SCC) and objective test results, as PwMS who report more cognitive complaints than can be confirmed by neuropsychological testing more often struggle with depression and fatigue.^4,5^ Conversely, some PwMS might notice cognitive changes in daily life before they become evident in objective assessments.^8^ Together, this suggests that the relationship between PROMs and objective cognitive function may vary among PwMS.

Despite recommendations for multifaceted cognitive screening in clinical care,^9^ an integrative approach to understanding these interrelated factors, rather than relying on univariate analyses, remains largely unexplored. To better understand the relationship between PROMs, including anxiety, depression, fatigue and SCC, and objectively assessed cognitive functioning, we explored symptom network analysis. This analysis examines the interactions among multiple symptoms rather than focusing on individual symptoms.^10^ In a network, nodes can represent PROMs or cognitive domains and edges represent associations between these at the group level.^10^ While network analysis has been applied to study self-reported symptoms in cancer and psychiatric disease,^11,12^ its application in MS remains understudied.

This study aimed to utilize network analysis to uncover correlational patterns of interrelatedness between objective cognitive functioning and PROMs in MS, to elucidate the subjective–objective discrepancy. We hypothesized that the relationship among these symptoms would differ between PwMS with and without cognitive impairments and those with and without SCC. To test this, we compared networks distinguishing between self-reported symptoms (i.e., SCC) and between objectively assessed impairment in IPS, the most common impairment in PwMS. Our objectives were to: (1) compute cognitive symptom networks in PwMS, (2) compare these networks between subgroups with less and more SCC, and (3) compare these networks between subgroups with and without IPS impairment. Through these comparisons, we sought to determine whether symptoms are more tightly interconnected in different subgroups of PwMS. Understanding these patterns could enhance clinical understanding, therapeutic interventions, and symptom management strategies, given the significant impact of cognitive impairment on quality of life and daily functioning.

## Materials and methods

### Participants

This study retrospectively evaluated cross-sectional data from eight observational studies performed between 2008 and 2023 at Amsterdam UMC location VUmc. The Medical Ethics Review Committee of Amsterdam UMC granted ethical approval, and all PwMS provided written informed consent. Table 1 summarizes cohort details and inclusion criteria, with previous publications listed in Supplementary Table 1. Participants were included if they met criteria for clinically definite MS or clinically isolated syndrome, completed PROMs, and underwent a neuropsychological assessment. PwMS with missing data were excluded (*n* = 209). If PwMS participated in multiple studies or visits (*n* = 43), only the first visit was included, resulting in a total of 703 PwMS eligible for analysis.

**Table 1.** Overview of the included cohorts, with their inclusion and exclusion criteria.

	*n* (% of total)	Inclusion criteria	Exclusion criteria
Cohorts			
1. Attention METC-number: 2014.377	86 (12.2)	• MS diagnosis according to the 2010-McDonald criteria (2) • 18–68 years of age • Ability to safely undergo an MRI examination • Screening for motor and visual skills	• History or presence of drug abuse • Neurological (other than MS) and psychiatric diseases • Relapse and/or steroid treatment 4 weeks prior to examination
2. Amsterdam MS cohort *General MS cohort* METC-number: 2002.140 *(P02.1381* *L), 2004.009 (P04.0142* *L)* *Longstanding MS cohort* *METC-number: 2010.336*	188 (26.7) 61/188 (32.4)	• MS diagnosis according to the 2010-McDonald criteria (2) • 18 years of age and older	• Neurological (other than MS) and psychiatric diseases • Relapse and/or steroid treatment 2 months prior to examination
	127/188 (67.6)	• MS diagnosis according to the 2010-McDonald criteria (2) • 18 years of age and older • Minimum disease duration of 10 years from onset	• Neurological (other than MS) and psychiatric diseases • Relapse and/or steroid treatment 6 weeks prior to examination
3. Fingolimod METC-number: 2014.418	45 (6.4)	• MS diagnosis according to the 2010-McDonald criteria (2) • PwMS with RRMS • 18–65 years of age • Ability to safely undergo an MRI examination • Screening for motor and visual skills	• Neurological (other than MS) and psychiatric diseases • Relapse and/or steroid treatment 4 weeks prior to examination
4. GABA & glutamate METC-number: 2017.380	49 (7.0)	• MS diagnosis according to the 2017-McDonald criteria (23) • PwMS with RRMS or SPMS • 18–65 years of age • Ability to safely undergo an MRI examination • Screening for motor and visual skills	• History or presence of drug abuse • Neurological (other than MS) and psychiatric diseases • Relapse and/or steroid treatment 4 weeks prior to examination
5. RemindMS^a^ METC-number: 2017.009	99 (14.1)	• MS diagnosis according to the 2010-McDonald criteria(2) • 18–65 years of age • Scoring ⩾ 23 on the Multiple Sclerosis Neuropsychological Questionnaire—Patient version (MSNQ-P)	• History/presence of psychosis and/or suicidal ideation • Inability to speak Dutch • Previous experience with the similar interventions • Physical or cognitive disabilities/ comorbidities/ treatments likely to cause interference
6. SOMSCOG^a^ METC-number: 2016.395	101 (14.4)	• MS diagnosis according to the 2017-McDonald criteria (23)	
7. Tecfidera METC-number: 2017.469	64 (9.1)	• MS diagnosis according to the 2017-McDonald criteria (23) • PwMS with RRMS • 18–65 years of age • Ability to safely undergo an MRI examination • Screening for motor and visual skills	• History or presence of drug abuse • Neurological (other than MS) and psychiatric diseases • Relapse and/or steroid treatment 4 weeks prior to examination • Participation in other studies using cognitive or physical training programs
8. Temprano METC-number: 2020.021	71 (10.1)	• MS diagnosis according to the 2017-McDonald criteria, within 1 year (23) • PwMS with RRMS • 18–65 years of age • Sufficient Dutch proficiency • Ability to safely undergo an MRI examination	• History or presence of drug abuse • Neurological (other than MS) and psychiatric diseases • Relapse and/or steroid treatment 4 weeks prior to examination • Participation in other studies using cognitive or physical training programs

MS: multiple sclerosis; PwMS: people with MS; MRI: Magnetic Resonance Imaging; RRMS: relapsing-remitting MS; SPMS: secondary progressive MS.

a Although the presence of an acute relapse was not an exclusion criteria for this study, no PwMS experienced a relapse when participating in the study.

Demographic and clinical characteristics were collected. Level of education was assessed according to the Verhage classification,^13^ and physical disability was assessed using the Expanded Disability Status Scale (EDSS).^14^

### Patient-reported outcome measures

Anxiety and depression symptoms were measured with the Hospital Anxiety and Depression Scale (HADS),^15^ and fatigue with the Checklist Individual Strength-20 Revised (CIS), including the subscales: subjective fatigue (CIS-subjective), concentration (CIS-concentration), motivation (CIS-motivation), and activity (CIS-activity).^16^ All PROMs were scaled (mean = 0, standard deviation (SD) =1) to allow for comparison between questionnaires, with higher scores indicating worse psychological functioning (listed in Supplementary Table 2).

SCC was assessed using multiple PROMs for different cohorts (the MS Neuropsychological Questionnaire-patient version (MSNQ),^17^ the Cognitive Failures Questionnaire (CFQ)^18^ and the Subjective Cognitive Performance Questionnaire (SCPQ)).^19^ Based on the *z*-scores obtained from each PROM, we constructed a single SCC variable. For three cohorts, PwMS completed two SCC PROMs, resulting in two *z*-scores. In such a case, the SCC was computed as the average of the two *z*-scores. Supplementary Table 3 details an explanation of this procedure.

### Neuropsychological assessment

Cognitive functioning was assessed using adapted versions of the Minimal Assessment of Cognitive Function in MS^20^ or the Brief Repeatable Battery of Neuropsychological Tests.^21^ Cognitive test scores from different cohorts were combined into six cognitive domains: attention, inhibition, IPS, verbal fluency, verbal memory, and visuospatial memory (see Supplementary Table 2).

Due to the statistical methods used, cognitive test scores were normed with two different approaches. First, scores were adjusted for age, sex, and education and transformed into domain-specific *z*-scores using normative data from Dutch healthy controls (*n* = 407).^22^ These data were used to report the sample characteristics and to define subgroups with and without IPS impairment, indicated by a *z*-score ⩽ –1.5 (third objective).^23^

Second, domain *z*-scores were calculated based on the PwMS sample mean and SD. These cognitive domains were used as input for the networks and were not corrected for demographics. This is because PROMs data are generally not corrected for demographic characteristics, and for statistical consistency, the input variables in a network should undergo the same scoring procedure.^24^

### Subgroups

**Figure 1.**
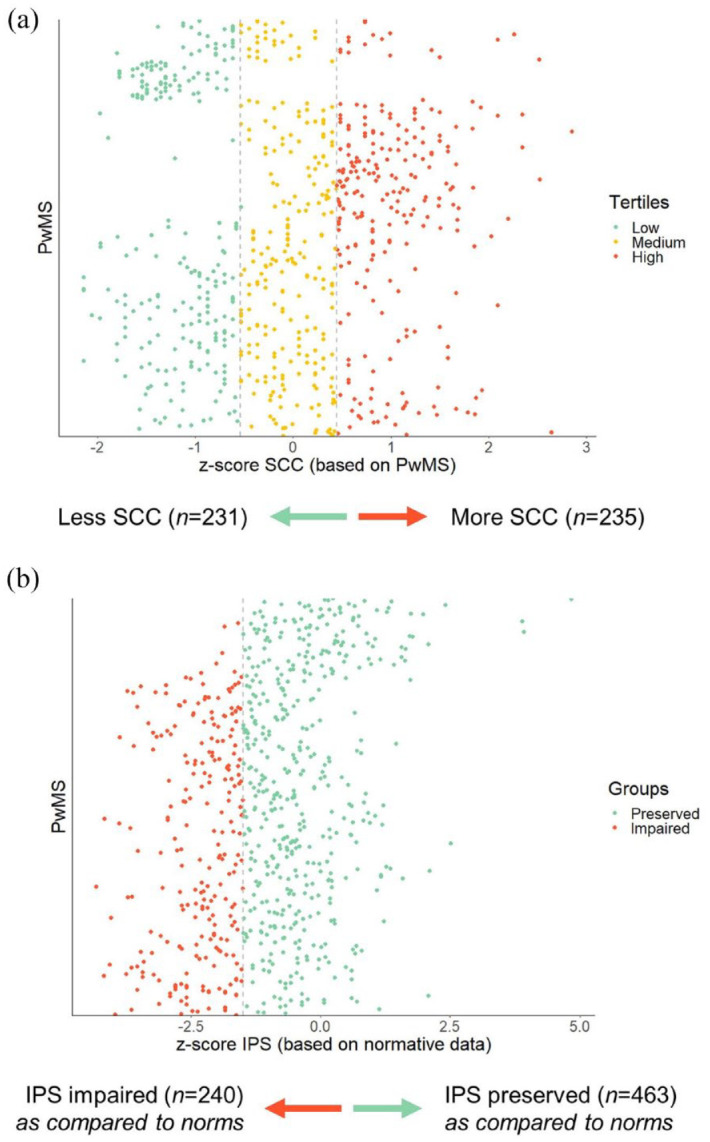
Constructing subgroups for network comparisons. (a) The *z*-scores were computed based on the group itself (PwMS), with higher scores indicating more problems. Based on tertiles, we divided the total sample into a “less SCC” group and a “more SCC” group. For this split, the middle tertile was left out of the analysis. (b) The *z*-scores were constructed based on normative data. Z-scores ⩽ –1.5 were considered impaired. PwMS: people with MS; SCC: self-reported cognitive complaints; IPS: information processing speed.

To explore the impact of SCC and IPS on the networks (objectives 2 and 3), we categorized PwMS into subgroups (see Figure 1).

#### SCC split

The entire sample was divided into tertiles based on SCC *z*-scores (see Figure 1(a)). PwMS in the lower tertile for SCC (less complaints) constituted the “*less SCC*” subgroup (*n* = 231), and PwMS in the higher tertile were part of the “*more SCC*” subgroup (more complaints, *n* = 235).

#### IPS split

We split the entire dataset into an “*IPS impaired*” subgroup (*n* = 240) and an “*IPS preserved*” subgroup (*n* = 463, see Figure 1(b)), using the *z*-scores based on normative data (described above, defining z-scores ⩽ –1.5 as impaired).

#### Sensitivity analyses

We split the complete dataset into tertiles based on IPS *z*-scores from PwMS (rather than normative data, thereby mirroring the SCC split). We compared the networks of PwMS between the lower and higher IPS tertiles. The dataset was also dichotomized based on sex. These sensitivity analyses are outlined in the Supplementary Materials (see Appendix A and B).

### Statistical analyses

Network and statistical analyses were conducted in RStudio (version 4.2.1),^25^ using the packages *bootnet*^24^ and *qgraph*.^26^ Normality of variables was checked by visually inspecting the histograms. Differences between subgroups were analyzed using independent samples *t*-tests for continuous variables and χ^2^-tests for categorical variables. An α-level of 0.05 was considered statistically significant. We followed the reporting guidelines for psychological network analyses in cross-sectional data.^27^

#### Computing networks

We introduce cognitive symptom networks,^12,24^ with nodes representing seven PROMs (sub)scales and six cognitive domains, connected by edges signifying regularized partial correlations between nodes at a group level.^10^ These *z*-scores of the cognitive domains were reversed to align with the PROMs (higher *z*-scores indicating worse cognitive performance). Detailed descriptions of the consecutive steps taken to construct the networks are included elsewhere.^11^ In short, networks were computed with Gaussian graphical models based on Spearman’s partial correlation matrices. Networks were regularized with EBICglasso with a tuning parameter set at 0.25 due to the explorative nature of this study.^24^ We present five networks: one comprising all PwMS with seven PROMs and six cognitive domains as nodes, and four subgroups networks based on SCC levels and IPS impairment. If a subgroup was dichotomized by SCC or IPS, the respective node was omitted from the network. We calculated node strength for each node per network, representing the sum of the edge weights connecting one node to others. If symptoms clustered within the network, these groups were called modules, representing symptoms that were more closely connected to each other.^28^

#### Comparing networks

To understand whether network density was different between subgroups, we calculated the global strength of the networks (objectives 2 and 3).^29^ Global strength is the average node strength of a network, and provides a measure of overall interconnectedness of nodes. Global strength was compared between networks with permutation-based network comparison tests using 2000 iterations.^29^ If there was a significant difference in global strength between subgroups, we split the network into a PROMs and a cognitive domains network and compared these networks between subgroups.

#### Stability and accuracy

Given the high number of estimated parameters, the stability of node strengths and the accuracy of estimated edges were evaluated (see Supplementary Table 4).^24^

## Results

### Participants

The sample of 703 PwMS included 71.3% females, with a mean age of 46.3 ± 11.2 years (see Table 2). Most PwMS had relapsing-remitting MS (RRMS; 79.8%), a median disease duration of 8.2 years (interquartile range: 2.9–16.9), a median EDSS of 3.5 (range: 0.0–8.0), and 57.5% used disease-modifying therapy (DMT). The domain-specific impairments were 34.1% for IPS, 23.6% for attention, 23.5% for inhibition, 16.9% for visuospatial memory, 16.4% for verbal memory, and 12.7% for verbal fluency.

**Table 2.** Demographic, disease-related, and psychological and cognitive characteristics of the total sample and per subgroups.

	Total sample		SCC split			IPS split		
	*n* = 703	% Missing	Less SCC *n* = 231	More SCC *n* = 235	*p*-value	IPS preserved *n* = 463	IPS impaired *n* = 240	*p*-value
Demographics								
Sex—female, *n* (%)	501 (71.3%)	0.0	163 (70.6%)	180 (76.6%)	0.140	337 (72.8%)	164 (68.3%)	0.216
Age—years, median (SD)	46.3 (11.2)	0.0	44.7 (11.0)	46.3 (10.6)	0.099	44.8 (11.3)	49.1 (10.5)	< 0.001*
Education—Verhage^a^, median (IQR)	6.0 (5.0–6.0)	0.3	6.0 (5.0–7.0)	6.0 (5.0–6.0)	0.085	6.0 (5.0–6.0)	6.0 (5.0–6.0)	0.107
Clinical functioning								
MS type		1.9			0.153			< 0.001*
RRMS—*n* (%)	561 (79.8%)		201 (87.0%)	183 (77.9%)		388 (83.8%)	173 (72.1%)	
SPMS—*n* (%)	83 (11.8%)		18 (7.8%)	30 (12.8%)		36 (7.8%)	47 (19.6%)	
PPMS—*n* (%)	42 (6.0%)		9 (3.9%)	14 (6.0%)		29 (6.3%)	13 (5.4%)	
CIS—*n* (%)	4 (0.6%)		1 (0.4%)	2 (0.9%)		3 (0.6%)	1 (0.4%)	
Disease duration—years, median (IQR)	8.2 (2.9–16.9)	2.4	6.3 (1.0–13.4)	9.0 (3.8–18.0)	< 0.001*	6.5 (1.5–14.0)	13.0 (5.9–19.9)	< 0.001*
EDSS^b^—median (range)	3.5 (0.0–8.0)	1.0	3.0 (0.0–7.5)	4.0 (1.0–8.0)	< 0.001*	3.0 (0.0–8.0)	4.0 (1.0–8.0)	< 0.001*
DMT use—yes, *n* (%)	404 (57.5%)	7.0	154 (66.7%)	132 (56.2%)	0.032*	282 (60.9%)	122 (50.8%)	0.050
PROMs								
CIS20-R sub-scale—mean (SD)	35.7 (12.1)	0.0	29.6 (12.1)	40.9 (10.6)	< 0.001*	34.8 (12.4)	37.5 (11.3)	0.004*
Severe fatigue| ⩾ 35, *n* (%)	398 (56.6%)		83 (35.9%)	176 (74.9%)	< 0.001*	250 (54.0%)	148 (61.7%)	0.052
HADS-D score—median (IQR)	4.0 (2.0–7.0)	0.0	2.0 (1.0–5.0)	6.0 (4.0–9.0)	< 0.001*	3.0 (1.0–7.0)	5.0 (3.0–8.0)	< 0.001*
Sig symptoms| ⩾ 8, *n* (%)	170 (24.2%)		34 (14.7%)	90 (38.3%)	< 0.001*	97 (21.0%)	73 (30.4%)	0.005*
HADS-A score—median (IQR)	6.0 (4.0–8.0)	0.0	5.0 (3.0–7.0)	8.0 (5.0–11.0)	< 0.001*	6.0 (4.0–8.0)	6.0 (4.0–9.0)	< 0.001*
Sig symptoms| ⩾ 8, *n* (%)	238 (33.9%)		51 (22.1%)	118 (50.2%)	< 0.001*	143 (30.9%)	95 (39.6%)	0.021*
SCC—mean (SD)	−0.05 (–0.06)	0.0	−1.2 (–1.1)	1.03 (0.9)	< 0.001*	−0.2 (1.0)	0.2 (1.0)	< 0.001*
Cognitive domains (*z*-scores compared to normative data)								
Attention—mean (SD)	−0.8 (1.2)	0.0	−0.05 (1.2)	−1.1 (1.2)	< 0.001*	−0.4 (1.0)	−1.6 (1.2)	< 0.001*
Impaired^c^—*n* (%)	166 (23.6%)		40 (17.3%)	78 (33.2%)	< 0.001*	56 (12.1%)	110 (45.8%)	< 0.001*
Inhibition—mean (SD)	−0.6 (1.4)	0.1^d^	−0.2 (1.3)	−1.1 (1.4)	< 0.001*	−0.3 (1.2)	−1.2 (1.4)	< 0.001*
Impaired^c^—*n* (%)	165 (23.5%)		33 (14.3%)	76 (32.3%)	< 0.001*	75 (16.2%)	90 (37.5%)	< 0.001*
IPS—mean (SD)	−1.0 (1.3)	0.0	−0.6 (1.2)	−1.3 (1.2)	< 0.001*	−0.3 (0.9)	−2.4 (0.7)	< 0.001*
Impaired^c^—*n* (%)	240 (34.1%)		52 (22.5%)	103 (43.8%)	< 0.001*	0 (0.0%)	240 (100%)	NA
Verbal fluency—mean (SD)	−0.7 (0.7)	0.3^e^	−0.7 (0.8)	−0.8 (0.8)	0.026*	−0.6 (0.7)	−1.1 (0.7)	< 0.001*
Impaired^c^—*n* (%)	89 (12.7%)		21 (9.1%)	40 (17.0%)	0.011*	32 (6.9%)	57 (23.8%)	< 0.001*
Verbal memory—mean (SD)	−0.5 (1.1)	0.3^e^	−0.3 (1.0)	−0.7 (1.1)	< 0.001*	−0.2 (0.9)	−1.1 (1.1)	< 0.001*
Impaired^c^—*n* (%)	115 (16.4%)		26 (11.3%)	45 (19.1%)	0.017*	36 (7.8%)	79 (32.9%)	< 0.001*
Visuospatial memory—mean (SD)	−0.5 (1.1)	0.0	−0.4 (1.1)	−0.5 (1.1)	0.242	−0.2 (1.0)	−0.9 (1.1)	< 0.001*
Impaired^c^—*n* (%)	119 (16.9%)		36 (15.6%)	39 (16.6%)	0.847	49 (10.6%)	70 (29.2%)	< 0.001*

SD: standard deviation; IQR: interquartile range; RRMS: relapsing-remitting MS; SPMS: secondary progressive MS; PPMS: primary progressive MS; CIS: clinically isolated syndrome; EDSS: Expanded Disability Status Scale; DMT: disease-modifying therapy; PROMs: patient-reported outcome measures; CIS20-R, Checklist Individual Strength-20 Revised; HADS-D, Hospital Anxiety and Depression Scale subscale Depression; Sig: significant; HADS-A, Hospital Anxiety and Depression Scale subscale Anxiety; SCC: self-reported cognitive complaints; IPS, information processing speed; NA: not applicable.

a Level of education is coded according to the Verhage classification.

b Assessed by a certified examiner, either face-to-face or through validated telephone assessment.

c Cognitive data in this table were corrected for age, sex, and educational level based on a normative sample of Dutch healthy controls, and considered impaired if *z*-score < –1.5.

d For one PwMS, comparison to normative data was unsuccessful as transformation to normal distribution (log-transformation) needed for comparison to normative data was not possible (time on Card III was faster than the average on Card I and II).

e Data for two PwMS was missing, as for comparison to normative data a correction with educational level was needed, and for these PwMS the level of education was not provided.

All significant p-values in bold.

* *p* < .05.

The SCC subgroups were similar regarding demographics and MS type. Compared to the “less SCC” subgroup, PwMS within the “more SCC” subgroup demonstrated a longer disease duration, higher EDSS, more frequent DMT use (range *p*-values < 0.001–0.032), and worse scores on all PROMs and cognitive domains (range *p*-values < 0.001–0.026), except for visuospatial memory (*p* = 0.242). Sex, educational level, DMT use, and the presence of severe fatigue were similar between the IPS subgroups (range *p*-values = 0.050–0.216). However, the “IPS impaired” subgroup scored worse on all other demographic, clinical, PROMs and cognitive domains (range *p*-values < 0.001–0.021).

### Cognitive symptom network

**Figure 2.**
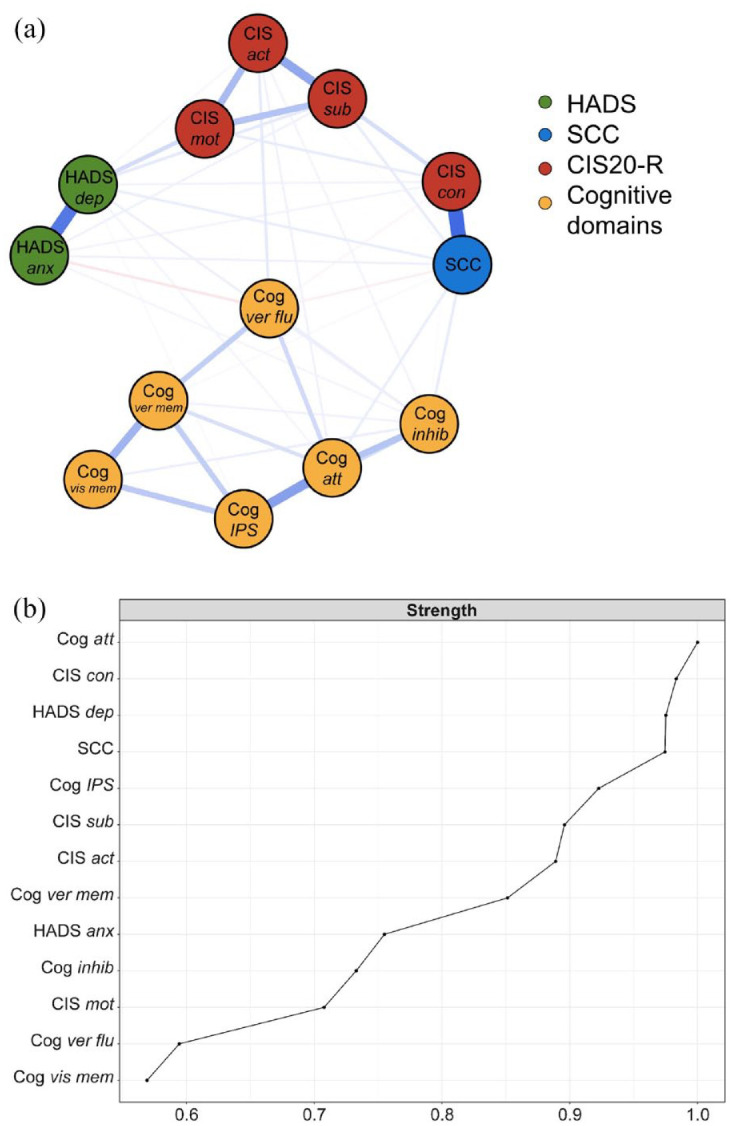
(a) The overall cognitive symptom network in PwMS. The colors of the nodes refer to the corresponding PROMs or cognitive domains. A blue edge indicates a positive relationship between the two nodes and a red edge a negative relationship. Edges were undirected and weighted and in the presented figures, edge width corresponds to the magnitude of the association. (b) Node strength is depicted with the cognitive domain “attention” showing the highest strength. HADS: Hospital Anxiety and Depression Scale; SCC: self-reported cognitive complaints; CIS20-R: Checklist Individual Strength (CIS)-20 Revised; Cog: cognitive domain; att: attention; inhib: inhibition; IPS: information processing speed; ver flu: verbal fluency; ver mem: verbal memory; vis mem: visuospatial memory; HADS anx: HADS anxiety subscale; HADS dep: HADS depression subscale; CIS sub: CIS-subjective; CIS con: CIS-concentration; CIS mot: CIS-motivation; CIS act: CIS-activity.

Figure 2 displays the cognitive symptom network of all 703 PwMS. The network comprised of 47 edges out of 78 possible edges (60.3%), connecting the 13 nodes. Visual inspection showed that PROMs and cognitive domain nodes primarily clustered into two modules, with weak links connecting these modules (see Figure 2(a), Supplementary Table 5). The nodes attention, CIS-concentration, HADS-D, and SCC had the highest node strength, indicating strong connections to other nodes (see Figure 2(b)). Each PROM and cognitive domain node was connected to at least one node from the other module. The strongest edges were present between SCC and CIS-concentration, HADS-D and HADS-A, attention and IPS, and CIS-activity and CIS-subjective (range edge weights = 0.586–0.340, see Supplementary Table 5). Stability checks indicated that node strength could be interpreted accurately (see Supplementary Figure 1).

### Comparing networks based on SCC

**Figure 3.**
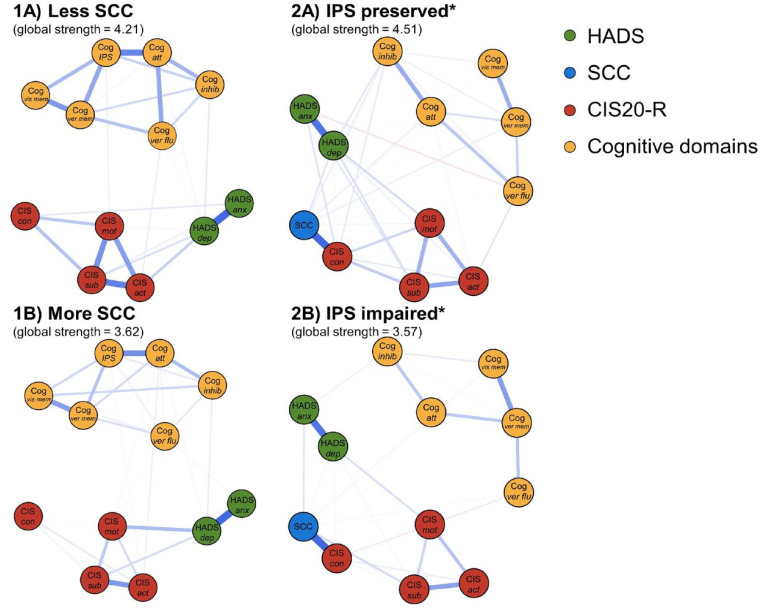
Comparisons of the cognitive symptom networks for the subgroups. The networks for the SCC subgroups can be found in (1A) and (1B). The networks for the IPS subgroups can be found in (2A) and (2B). The colors of the nodes refer to the corresponding PROMs or cognitive domains. A blue line indicates a positive relationship between the two nodes, and a red line indicates a negative relationship. Edges were undirected and weighted, and in the presented figures, edge width corresponds to the magnitude of the association. HADS: Hospital Anxiety and Depression Scale; SCC: self-reported cognitive complaints; CIS20-R: Checklist Individual Strength (CIS)-20 Revised; Cog: cognitive domain; att: attention; inhib: inhibition; IPS: information processing speed; ver flu: verbal fluency; ver mem: verbal memory; vis mem: visuospatial memory; HADS anx: HADS anxiety subscale; HADS dep: HADS depression subscale; CIS sub: CIS-subjective; CIS con: CIS-concentration; CIS mot: CIS-motivation; CIS act: CIS-activity. *Global strength of the network is higher for the network of PwMS with preserved IPS, compared to impaired IPS (*p* = 0.001).

Figure 3(1A) and (1B) depict the networks for the “less SCC” and “more SCC” subgroups, respectively. Global strength was not significantly different between these (4.21 versus 3.62, respectively, *p* = 0.140), indicating similar overall interconnectedness of the nodes in both networks. Supplementary Figures 2 and 3 show details on node strength, stability, and edge accuracy.

### Comparing networks based on IPS impairment

**Figure 4.**
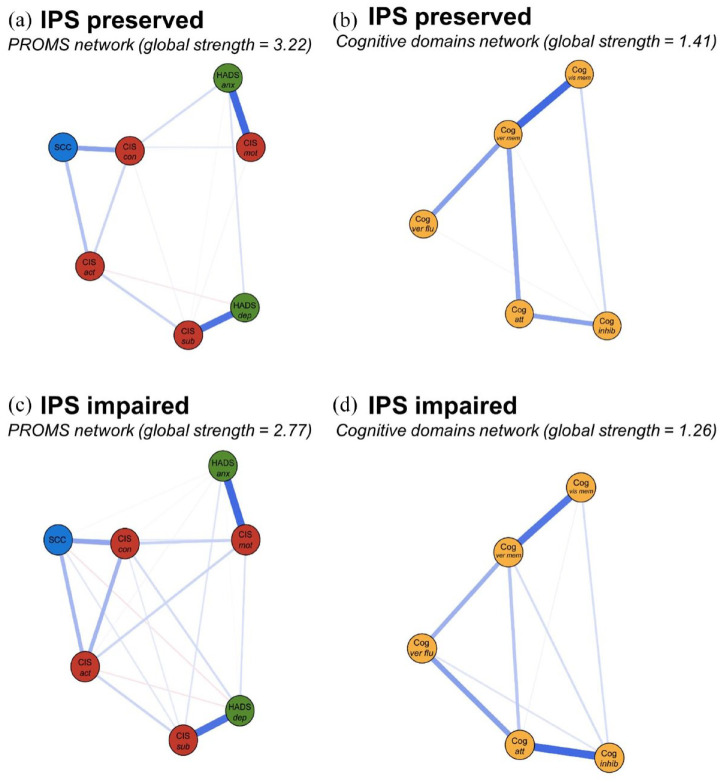
Comparisons of the PROMs and objective cognitive modules for “IPS preserved” PwMS (a and b, respectively) and the “IPS impaired” PwMS (c and d, respectively). The colors of the nodes refer to the corresponding PROMs or cognitive domain (yellow). Nodes on depression and anxiety are depicted in green, nodes on fatigue in red, and SCC is depicted in blue. A blue edge indicates a positive relationship between the two nodes and a red edge a negative relationship. Edges were undirected and weighted and in the presented figures, edge width corresponds to the magnitude of the association. For each network, the global strength is also indicated. Cog: cognitive domain; att: attention; inhib: inhibition; ver flu: verbal fluency; ver mem: verbal memory; vis mem: visuospatial memory; HADS anx: Hospital Anxiety and Depression Scale anxiety subscale; HADS dep: Hospital Anxiety and Depression Scale depression subscale; CIS: Checklist Individual Strength-20 Revised; CIS sub: CIS-subjective; CIS con: CIS-concentration; CIS mot: CIS-motivation; CIS act: CIS-activity.

We found a lower global strength of the “IPS impaired” network compared to the “IPS preserved” network (3.57 versus 4.51, respectively, *p* = 0.001), indicating lower correlations among PROMs and cognitive domains in PwMS with impaired IPS (see Figure 3(2A) and (2B)). To further understand these results, we compared the global strength of separate cognitive and separate PROMs networks between the subgroups (see Figure 4), but no differences in global strength were found for these networks (*p* = 0.080, *p* = 0.250, respectively).

Since the difference in global strength was observed in the overall network only, not within the separate PROMs and cognitive networks, the difference in global strength of the networks between PwMS with and without IPS impairment may be due to weaker associations connecting PROMs and cognitive domains (although this was not specifically tested). Caution is warranted when interpreting the global strength of the PROMs network, as the nodes’ stability is below the preferred threshold (see Supplementary Figures 4–9).

In a post hoc analysis of RRMS PwMS, we confirmed previous results: global strength of the “IPS preserved” network was 4.84 (*n* = 391) versus 3.03 for the “IPS impaired network” (*n* = 170; *p* = 0.010). No significant differences were found between groups for the separate cognitive (*p* = 0.723) or PROMs networks (*p* = 0.495).

### Sensitivity analyses

No differences in global network strength were found between lower and higher IPS functioning (*p* = 0.080, see Supplementary Appendix A) or between females and males (*p* = 0.470, see Supplementary Appendix B).

## Discussion

This study aimed to investigate the complex interplay between self-reported symptoms and objectively assessed cognitive functioning in PwMS by quantifying a cognitive symptom network based on PROMs (anxiety, depression, fatigue, and SCC) and cognitive domains. In this network, we observed clustering of nodes into two modules: a PROMs module and an objective cognitive module, connected by numerous weak edges. Particularly attention, fatigue (concentration-subscale), depression, and SCC were highly connected within the network. Second, we aimed to better understand the cognitive subjective–objective discrepancy in MS. Therefore, we studied how SCC and IPS impacted the networks, by comparing the global strength of the networks among subgroups. Networks for PwMS with different levels of SCC were similar. Interestingly, PwMS with IPS impairments demonstrated a lower global strength of the network compared to those with preserved IPS, indicating that PROMs and cognitive domains were less tightly interrelated for PwMS with impaired IPS.

Our first objective was to compute a cognitive symptom network and explore its potential for studying symptom interrelatedness in MS. The network showed distinct modules for PROMs and objectively assessed cognitive domains, supporting the expected subjective–objective discrepancy.^4,5^ Specifically, SCC was mainly connected to other PROMs, a pattern also observed in networks of psychiatric populations.^12^ Another study using network analysis in early RRMS, which also included physical and imaging outcomes, found self-reported fatigue to be more strongly associated with depression and physical disability compared to cognitive and imaging outcomes.^30^ In our network, we observed a central role for attention, fatigue (concentration subscale), depression, and SCC. Attention has been linked to symptom awareness and preoccupation,^31^ potentially explaining its central role in our network. Furthermore, fatigue (concentration-subscale) and SCC specifically address self-reported aspects related to cognitive functioning, such as concentration and attention. This specificity makes their central role in this cognitive symptom network unsurprising. In addition, a meta-analysis demonstrated that heightened depressive symptomatology is strongly associated with increased cognitive difficulties.^32^ The identified central nodes align with prior literature, supporting the viability of this multi-dimensional approach. While central nodes could help select intervention targets,^10^ understanding causal interconnections requires longitudinal study designs.^33^ Nevertheless, the cross-sectional networks presented in our study still offer valuable insights into the co-occurrence of symptoms,^34^ which is crucial for understanding complex and heterogeneous diseases like MS.

Second, we aimed to shed light on the subjective–objective cognitive discrepancy in PwMS. We found lower symptom interrelatedness for PwMS with impaired IPS compared to those with preserved IPS. In literature, self-reported cognitive measures primarily correlate with depression and fatigue, instead of cognitive test scores.^17^ Similarly, a study found a stronger correlation between actual test performance and estimations about performance, as opposed to perceptions of daily cognitive functioning, with the latter not reaching statistical significance.^35^ The subjective–objective discrepancy has gained renewed focus due to the growing importance of cognitive screening and monitoring tools for PwMS.^1^ This discrepancy is often studied using univariate associations among these variables, constructing independent regression models for SCC or objective measures, or calculating/predicting discrepancy scores between SCC and objective cognitive functioning, categorizing PwMS as “under” or “over” estimators (facing statistical challenges such as multicollinearity when building prediction models).^4,5,8^ “Under” estimators (with more self-reported problems than cognitive deficits, leading them to underestimate their performance) comprised the largest proportions of PwMS (39%–43%),^4,5^ scoring higher on depression and fatigue compared to other groups,^4,5^ with cognitive fatigue^5^ and estimated premorbid cognitive functioning^8^ being key predictors of these discrepancy scores. Our multi-dimensional approach suggests that the subjective–objective discrepancy becomes more pronounced with increasing objective IPS deficits, indicating a nonlinear relationship between subjective and objective outcomes. The finding of lower symptom interrelatedness with worse IPS is particularly intriguing, as one would expect greater levels of depression, anxiety, and fatigue to go hand in hand with experiencing more cognitive deficits.^1^

In clinical research settings, these insights should prompt a careful reevaluation of subjective and objective cognition, given the increasing challenge of accurately determining the specific (cognitive) deficits in PwMS based solely on self-reported information. Our findings confirm that symptomatology worsens for PwMS with impaired IPS (based on our sample characterization), but also reveal different patterns of symptom co-occurrence for PwMS with preserved and impaired IPS. It may be hypothesized that individuals with impaired IPS may have reduced accuracy in self-assessing their cognitive functioning due to broader deficits, affecting their ability to perceive and report accurately. The co-occurrence between psychological and cognitive symptoms appears more widespread in PwMS with impaired IPS. Speculatively, as cognitive deficits escalate, other symptoms tend to become more widespread. Therefore, it is crucial to carefully monitor the emerging symptoms individuals may experience.

A strength of this study is its relatively large sample, including retrospective data from eight different cohorts. However, this also posed challenges in constructing networks. For instance, only 125 progressive PwMS (17.8%) were included, preventing a network split based on MS type. Limited data on disease-specific information (such as lesion load or the use of specific DMTs) restricted our ability to investigate those variables within the network or between relevant groups, highlighting potential avenues for future research. Combining multiple cohorts resulted in clustered data, a limitation that we addressed by applying bootstrapping procedures to ensure more robust estimates.^24^ Furthermore, we were unable to include working memory or cognitive flexibility, which are acknowledged to be affected in MS.^1^ For SCC, we had to utilize various questionnaires across different cohorts. This limitation is somewhat mitigated by existing literature demonstrating a large correlation between the MSNQ and the CFQ.^17^

In conclusion, we studied the interrelatedness between PROMs and objective cognitive domains in PwMS using network analysis. We found that, within the cognitive symptom network, PROMs and cognitive domains cluster separately but are still represented as one network. The finding of lower network interrelatedness for PwMS with impaired IPS, and not SCC, might suggest that the relation between subjectively and objectively measured symptoms does not follow a linear continuum but is dependent on the cognitive status of the PwMS. In PwMS with impaired IPS, patterns of psychological and cognitive symptoms are more widespread, contributing to the heterogeneity of clinical presentations as the disease progresses.

## Supplemental Material

sj-docx-1-msj-10.1177_13524585241302173 – Supplemental material for Understanding the complex network of objectively assessed cognition and self-reported psychological symptoms in people with multiple sclerosis Supplemental material, sj-docx-1-msj-10.1177_13524585241302173 for Understanding the complex network of objectively assessed cognition and self-reported psychological symptoms in people with multiple sclerosis by Maureen van Dam, Jantine G Röttgering, Ilse M Nauta, Brigit A de Jong, Martin Klein, Menno M Schoonheim, Bernard MJ Uitdehaag, Hanneke E Hulst and Linda Douw in Multiple Sclerosis Journal

## References

[1] Benedict RHB Amato MP DeLuca J , et al. Cognitive impairment in multiple sclerosis: Clinical management, MRI, and therapeutic avenues. Lancet Neurol 2020; 19(10): 860–871. 32949546 10.1016/S1474-4422(20)30277-5 PMC10011205

[2] Wojcik C Fuchs TA Tran H , et al. Staging and stratifying cognitive dysfunction in multiple sclerosis. Mult Scler 2022; 28(3): 463–471. 33951975 10.1177/13524585211011390

[3] Benedict RH Zivadinov R . Predicting neuropsychological abnormalities in multiple sclerosis. J Neurol Sci 2006; 245: 67–72. 16626751 10.1016/j.jns.2005.05.020

[4] Hughes AJ Bhattarai JJ Paul S , et al. Depressive symptoms and fatigue as predictors of objective-subjective discrepancies in cognitive function in multiple sclerosis. Mult Scler Relat Disord 2019; 30: 192–197. 30797133 10.1016/j.msard.2019.01.055 PMC7282884

[5] Davenport L Cogley C Monaghan R , et al. Investigating the association of mood and fatigue with objective and subjective cognitive impairment in multiple sclerosis. J Neuropsychol 2022; 16(3): 537–554. 35765743 10.1111/jnp.12283

[6] Boeschoten RE Braamse AM Beekman AT , et al. Prevalence of depression and anxiety in multiple sclerosis: A systematic review and meta-analysis. J Neurol Sci 2017; 372: 331–341. 28017241 10.1016/j.jns.2016.11.067

[7] Krupp L . Fatigue is intrinsic to multiple sclerosis (MS) and is the most commonly reported symptom of the disease. Mult Scler 2006; 12(4): 367–368. 16900749 10.1191/135248506ms1373ed

[8] Stein C O’Keeffe F McManus C , et al. Premorbid cognitive functioning influences differences between self-reported cognitive difficulties and cognitive assessment in multiple sclerosis. J Neuropsychol 2024; 18(1): 47–65. 37212461 10.1111/jnp.12327

[9] Westergaard K Skovgaard L Magyari M , et al. Patient perspectives on patient-reported outcomes in multiple sclerosis treatment trajectories: A qualitative study of why, what, and how? Mult Scler Relat Disord 2022; 58: 103475. 34995975 10.1016/j.msard.2021.103475

[10] Borsboom D . A network theory of mental disorders. World Psychiatry 2017; 16: 5–13. 28127906 10.1002/wps.20375 PMC5269502

[11] Röttgering JG Varkevisser TMCK Gorter M , et al. Symptom networks in glioma patients: Understanding the multidimensionality of symptoms and quality of life. J Cancer Surviv 2024; 18(3): 1032–1041. 36922442 10.1007/s11764-023-01355-8 PMC11082018

[12] Chavez-Baldini U Nieman DH Keestra A , et al. The relationship between cognitive functioning and psychopathology in patients with psychiatric disorders: A transdiagnostic network analysis. Psychol Med 2023; 53(2): 476–485. 34165065 10.1017/S0033291721001781 PMC9899564

[13] Verhage F . Intelligentie en leeftijd: Onderzoek bij Nederlanders van twaalf tot zevenenzeventig jaar. Assen: Van Gorcum, 1964.

[14] Lechner-Scott J Kappos L Hofman M , et al. Can the Expanded Disability Status Scale be assessed by telephone? Mult Scler J 2003; 9: 154–159. 10.1191/1352458503ms884oa 12708811

[15] Zigmond AS Snaith RP . The hospital anxiety and depression scale. Acta Psychiatr Scand 1983; 67: 361–370. 6880820 10.1111/j.1600-0447.1983.tb09716.x

[16] Vercoulen JH Swanink CM Fennis JF , et al. Dimensional assessment of chronic fatigue syndrome. J Psychosom Res 1994; 38: 383–392. 7965927 10.1016/0022-3999(94)90099-x

[17] Benedict RH Munschauer F Linn R , et al. Screening for multiple sclerosis cognitive impairment using a self-administered 15-item questionnaire. Mult Scler 2003; 9(1): 95–101. 12617275 10.1191/1352458503ms861oa

[18] Merckelbach H Muris P Nijman H , et al. Self-reported cognitive failures and neurotic symptomatology. Personal Individ Differ 1996; 20: 715–724.

[19] Stewart AL Ware J Sherbourne CD , et al. Psychological distress/well-being and cognitive functioning measures. In: Stewart AL Ware JE (eds) Measuring functioning and well-being: The medical outcomes study approach. London: Duke University Press, 1992, pp. 102–142.

[20] Benedict RH Cookfair D Gavett R , et al. Validity of the minimal assessment of cognitive function in multiple sclerosis (MACFIMS). J Int Neuropsychol Soc 2006; 12: 549. 16981607 10.1017/s1355617706060723

[21] Broeders TAA Douw L Eijlers AJC , et al. A more unstable resting-state functional network in cognitively declining multiple sclerosis. Brain Commun 2022; 4(2): fcac095. 10.1093/braincomms/fcac095 PMC9128379 35620116

[22] van Dam M de Jong BA Willemse EAJ , et al. A multimodal marker for cognitive functioning in multiple sclerosis: The role of NfL, GFAP and conventional MRI in predicting cognitive functioning in a prospective clinical cohort. J Neurol 2023; 270(8): 3851–3861. 37101095 10.1007/s00415-023-11676-4 PMC10344976

[23] Fischer M Kunkel A Bublak P , et al. How reliable is the classification of cognitive impairment across different criteria in early and late stages of multiple sclerosis? J Neurol Sci 2014; 343: 91–99. 24950898 10.1016/j.jns.2014.05.042

[24] Epskamp S Borsboom D Fried EI . Estimating psychological networks and their accuracy: A tutorial paper. Behav Res Methods 2018; 50(1): 195–212. 28342071 10.3758/s13428-017-0862-1 PMC5809547

[25] RStudio Team. RIDEfR, Boston, MA: RStudio, PBC, 2020.

[26] Epskamp S Cramer AO Waldorp LJ , et al. Qgraph: Network visualizations of relationships in psychometric data. J Stat Softw 2012; 48: 1–18.

[27] Burger J Isvoranu A Lunansky G , et al. Reporting standards for psychological network analyses in cross-sectional data. Psychol Methods 2020; 28(4): 806–824. 10.1037/met0000471 35404629

[28] Stam CJ . Modern network science of neurological disorders. Nat Rev Neurosci 2014; 15(10): 683–695. 25186238 10.1038/nrn3801

[29] van Borkulo CD van Bork R Boschloo L , et al. Comparing network structures on three aspects: A permutation test. Psychol Methods 2023; 28(6): 1273–1285. 35404628 10.1037/met0000476

[30] Chang YT Kearns PKA Carson A , et al. Network analysis characterizes key associations between subjective fatigue and specific depressive symptoms in early relapsing-remitting multiple sclerosis. Mult Scler Relat Disord 2023; 69: 104429. 36493562 10.1016/j.msard.2022.104429

[31] Solem S Hagen R Wang CE , et al. Metacognitions and mindful attention awareness in depression: A comparison of currently depressed, previously depressed and never depressed individuals. Clin Psychol Psychother 2017; 24(1): 94–102. 26450662 10.1002/cpp.1983

[32] Altieri M Cerciello F Gallo A , et al. The relationship between depression and cognitive performance in multiple sclerosis: A meta-analysis. Clin Neuropsychol 2023; 38(1): 21–41. 36964744 10.1080/13854046.2023.2192963

[33] Fried EI Cramer AOJ . Moving forward: Challenges and directions for psychopathological network theory and methodology. Perspect Psychol Sci 2017; 12(6): 999–1020. 28873325 10.1177/1745691617705892

[34] Bos FM Snippe E de Vos S , et al. Can we jump from cross-sectional to dynamic interpretations of networks implications for the network perspective in psychiatry. Psychother Psychosom 2017; 86(3): 175–177. 28490028 10.1159/000453583 PMC5516409

[35] Middleton LS Denney DR Lynch SG , et al. The relationship between perceived and objective cognitive functioning in multiple sclerosis. Arch Clin Neuropsychol 2006; 21(5): 487–494. 16879944 10.1016/j.acn.2006.06.008

