# Supplementary material for: Understanding the complex network of objectively assessed cognition and self-reported psychological symptoms in people with multiple sclerosis
Source: Mult Scler. 2024 Dec 9;31(1):93–106. doi: 10.1177/13524585241302173 (PMC11720265; doi:10.1177/13524585241302173)
Supplement: sj-docx-1-msj-10.1177_13524585241302173 – Supplemental material for Understanding the complex network of objectively assessed cognition and self-reported psychological symptoms in people with multiple sclerosis [file sj-docx-1-msj-10.1177_13524585241302173.docx]

# Supplementary materials

**Title:** Understanding the complex network of objectively assessed cognition and self-reported psychological symptoms in people with multiple sclerosis

**Authors:** M. van Dam^1,2^, J.G. Röttgering^3,4^, I.M. Nauta^5^, B.A. de Jong^5^, M. Klein^3,4^, M.M. Schoonheim^1^, B.M.J. Uitdehaag^5^, H.E. Hulst^2,6^ & L. Douw^1^

Author affiliations:

^1^ MS Center Amsterdam, Anatomy & Neurosciences, Vrije Universiteit Amsterdam, Amsterdam Neuroscience, Amsterdam UMC Location VUmc, Amsterdam, The Netherlands. ^2^ Leiden University, Institute of Psychology, Health, Medical and Neuropsychology Unit, Wassenaarseweg 52, Leiden, The Netherlands. ^3^ Amsterdam UMC Location Vrije Universiteit Amsterdam, Medical Psychology, De Boelelaan 1117, Amsterdam, The Netherlands. ^4^ Cancer Center Amsterdam, Brain Tumor Center, Amsterdam, The Netherlands. ^5^ MS Center Amsterdam, Neurology, Vrije Universiteit Amsterdam, Amsterdam Neuroscience, Amsterdam UMC Location VUmc, Amsterdam, The Netherlands. ^6^ Leiden Institute for Brain and Cognition, Leiden, The Netherlands

**Corresponding author:** M. van Dam (ORCID-ID: 0000-0002-4639-5138), Amsterdam UMC, location VUmc, Department of Anatomy and Neurosciences, MS Center Amsterdam, PO Box 7057, 1007 MB Amsterdam, The Netherlands; E-mail: [m.vandam2@amsterdamumc.nl](mailto:m.vandam2@amsterdamumc.nl)

Table of Contents

[**Supplementary Table 1**. Overview of the included cohorts, with their corresponding references. 4](#_Toc151377388)

[**Supplementary Table 2**. Overview of the included tests per cognitive factor, the corresponding test scores and the number of cohorts (eight in total) that included the specific test in their design. 6](#_Toc151377389)

[**Supplementary Table 3**. Background on self-reported cognitive complaints (SCC). 7](#_Toc151377389)

[**Supplementary Table 4**. The applied bootstrapping procedure to quantify the stability and accuracy of the networks.. 8](#_Toc151377389)

[**Supplementary Table 5**. Regularized partial correlation matrix of the full cognitive symptom network. 9](#_Toc151377389)

[**Supplementary Figure 1.** The stability of the nodes and the accuracy of the edge weights for the overall cognitive symptom network (*n* = 703). 10](#_Toc151377389)

[**Supplementary Figure 2.** The strength and the stability of the nodes and the accuracy of the edge weights for the cognitive symptom network within the ‘less SCC’ subgroup (*n* = 231)... 12](#_Toc151377389)

[**Supplementary Figure 3.** The strength and the stability of the nodes and the accuracy of the edge weights for the cognitive symptom network within the ‘more SCC’ subgroup (*n* = 235)... 14](#_Toc151377389)

[**Supplementary Figure 4.** The strength and the stability of the nodes and the accuracy of the edge weights for the cognitive symptom network within the ‘IPS preserved’ subgroup (*n* = 463)... 16](#_Toc151377389)

[**Supplementary Figure 5.** The strength and the stability of the nodes and the accuracy of the edge weights for the PROMS network within the ‘IPS preserved’ subgroup (*n* = 463).... 18](#_Toc151377389)

[**Supplementary Figure 6.** The strength and the stability of the nodes and the accuracy of the edge weights for the cognitive domains network within the ‘IPS preserved’ subgroup (*n* = 463)..... 20](#_Toc151377389)

[**Supplementary Figure 7.** The strength and the stability of the nodes and the accuracy of the edge weights for the cognitive symptom network within the ‘IPS impaired’ subgroup (*n* = 240)..... 22](#_Toc151377389)

[**Supplementary Figure 8.** The strength and the stability of the nodes and the accuracy of the edge weights for the PROMS network within the ‘IPS impaired’ subgroup (*n* = 240)..... 24](#_Toc151377389)

[**Supplementary Figure 9.** The strength and the stability of the nodes and the accuracy of the edge weights for the cognitive domains network within the ‘IPS impaired’ subgroup (*n* = 240).... 26](#_Toc151377389)

**Appendix A.** [**Supplementary Figure 10.** The cognitive symptom networks in PwMS – split based on IPS functioning in PwMS.... 28](#_Toc151377389)

**Appendix A.** [**Supplementary Figure 11.** The strength and the stability of the nodes and the accuracy of the edge weights for the cognitive symptom network within the ‘lower IPS tertile’ subgroup (indicating better performance, *n* = 232)..... 30](#_Toc151377389)

**Appendix A.** [**Supplementary Figure 12.** The strength and the stability of the nodes and the accuracy of the edge weights for the cognitive symptom network within the ‘higher IPS tertile’ subgroup (indicating poorer performance, *n* = 234)...... 32](#_Toc151377389)

**Appendix B.** [**Supplementary Figure 13.** The cognitive symptom networks in PwMS – split based on sex..... 34](#_Toc151377389)

**Appendix B.** [**Supplementary Figure 14.** The strength and the stability of the nodes and the accuracy of the edge weights for the cognitive symptom network within females (*n* = 501)..... 36](#_Toc151377389)

**Appendix B.** [**Supplementary Figure 15.** The strength and the stability of the nodes and the accuracy of the edge weights for the cognitive symptom network within males (*n* = 202)..... 38](#_Toc151377389)

[**Supplemental references**..... 40](#_Toc151377389)

# Supplementary Table 1. Overview of the included cohorts, with their corresponding references.

|  | ***n***  **(% of total)** |
| --- | --- |
| ***Cohorts*** |  |
| 1. Attention (1)  METC-number: 2014.377 | 86 (12.2) |
| 2. Amsterdam MS cohort (3-20)  *General MS cohort*  METC-number: 2002.140 *(P02.1381L), 2004.009 (P04.0142L)*  *Longstanding MS cohort*  *METC-number: 2010.336* | 188 (26.7)  61/188 (32.4) |
|  | 127/188 (67.6) |
| 3. Fingolimod (21)  METC-number: 2014.418 | 45 (6.4) |
| 4. GABA & glutamate (22)  METC-number: 2017.380 | 49 (7.0) |
| 5. RemindMS (24, 25)  METC-number: 2017.009 | 99 (14.1) |
| 6. SOMSCOG (26, 27)  METC-number: 2016.395 | 101 (14.4) |
| 7. Tecfidera  METC-number: 2017.469 | 64 (9.1) |
| 8. Temprano  METC-number: 2020.021 | 71 (10.1) |

*Abbrevations: MS = Multiple Sclerosis ; RRMS = Relapsing-Remitting MS.*

# Supplementary Table 2. Overview of the included tests per cognitive factor, the corresponding test scores and the number of cohorts (eight in total) that included the specific test in their design.

|  | **Test** | **Corresponding test scores and subscales** | **# of Cohorts** |
| --- | --- | --- | --- |
| ***Patient-reported outcome measures*** | |  |  |
| Anxiety | - Hospital Anxiety and Depression Scale | - Subscale anxiety | 8 |
| Depression | - Hospital Anxiety and Depression Scale | - Subscale depression | 8 |
| Fatigue | - Checklist Individual Strength-20 Revised | - Subscale subjective fatigue - Subscale concentration - Subscale motivation - Subscale activity | 8 |
| SCC | - Multiple Sclerosis Neuropsychological Questionnaire | - Total score | 4 |
|  | - Cognitive Failure Questionnaire | - Total score | 4 |
|  | - Subjective Cognitive Performance Questionnaire | - Total score | 3 |
| ***Cognitive domains*** | |  |  |
| Verbal memory | - California Verbal Learning Test – Version 2 | - Direct recall - Delayed recall - Recognition | 7 |
|  | - Selective Reminding Test | - Long-term storage 1 - Long-term retrieval sum - Short-term retrieval sum - Delayed recall | 1 |
| Visuospatial memory | - Location Learning Test | - Sum of displacement scores (five trials in total) | 4 |
|  | - Brief Visuospatial Memory Test – Revised | - Direct recall - Delayed recall - Recognition | 5 |
|  | - Spatial Recall Test | - Direct recall - Delayed recall | 1 |
| Information processing speed | - Symbol Digit Modalities Test | - Total of correct responses - reading subscale | 4 |
|  | - Letter Digit Substitution Test | - Total of correct responses - reading subscale | 6 |
| Attention | - Stroop Color-Word Test | - Time to complete card I - Time to complete card II | 8 |
| Executive functioning – Inhibition | - Stroop Color-Word Test | - Time to complete card III – (Time to complete card I + card II) | 8 |
| Executive functioning – Verbal fluency | - Controlled Oral Word Association Test | - Trial 1 (letter D) - Trial 2 (letter A) - Trial 3 (letter T) | 5 |
|  | - Word List Generation | - Trial 1 (animals) - Trial 2 (professions) - Trial 3 (m-words) | 5 |

**Supplementary Table 3.** Background on self-reported cognitive complaints (SCC).

| **Procedure** | | | **Description** | | | | | | |
| --- | --- | --- | --- | --- | --- | --- | --- | --- | --- |
| Construction of the self-reported cognitive complaints (SCC) node | | | ***Included questionnaires:***  In our sample, SCC was measured with three different PROMS: the MSNQ, the CFQ, and the SCPQ.  ***Availability:***  A total of 5 cohorts (i.e., Amsterdam MS cohort, GABA & Glutamate, SOMSCOG, Tecfidera and Temprano) had at least one of the abovementioned PROMS included (see the list on the distribution in the lower part of this Table). A total of 3 cohorts (i.e., Attention, Fingolimod and RemindMS) had two PROMS included, also listed in the panel below.  ***Calculation of z-scores:***  Based on the available data per PROM, the mean and standard deviation (SD) of the PwMS was calculation, after which raw scores were transferred into *z*-scores. The specific sample size per PROM, along with the mean and SD is included below. This *z*-score was called the SCC node. In case a PwMS had filled-out two PROMS, and had therefore two *z*-scores, the average of the two *z*-scores was computed for the SCC.  ***Check SCC node:***  We calculated the correlation between the final SCC-node (*z*-score) and the *z*-scores of the individual PROMS, as an indication of overlap. These results are included in the panel down blow. For the PwMS that had filled-out two PROMS, we also correlated both *z*-scores (and their corresponding sample size, as not all PwMS filled out the two PROMS). | | | | | | |
|  | |  | **Descriptives** | | | **Correlational analyses**  **(using *z*-scores)** | | | |
|  | | **Cohorts** | ***n*** | **Mean raw score** | **SD raw score** | **SCC** | **MSNQ** | **CFQ** | **SCPQ** |
| ***PROMS*** | |  |  |  |  |  |  |  |  |
|  | MSNQ | - Amsterdam MS cohort - RemindMS - SOMSCOG - Temprano | 449 | 26.3 | 11.4 | 0.990 | - | 0.480 (*n*=99) | NA |
|  | CFQ | - Attention - Fingolimod - RemindMS - Tecfidera | 292 | 37.3 | 21.3 | 0.967 | - | - | 0.839 (*n*=129) |
|  | SCPQ | - Attention - Fingolimod - GABA & glutamate | 180 | 11.4 | 5.8 | 0.973 | - | - | - |

*Abbreviations: SCC = Self-reported Cognitive Complaints; PROMS = Patient-Reported Outcome Measures; MSNQ = Multiple Sclerosis Neuropsychological Questionnaire; CFQ = Cognitive Failure Questionnaire; SCPQ = Subjective Cognitive Performance Questionnaire; PwMS = People with MS; NA = Not Available.*

**Supplementary Table 4.** The applied bootstrapping procedure to quantify the stability and accuracy of the networks.

| **Procedure** | **Description** |
| --- | --- |
| Stability of the nodes | To assess the stability of the strength of the nodes, we performed a case-dropping bootstrap with 1000 iterations (28). We computed correlation stability coefficients for each network as a measure of node stability. This measure should at least be ≥0.25, and preferably ≥0.50 (28). |
| Accuracy of edge weights | To assess the accuracy of the edge estimates for each of the networks, we estimated 95% confidence intervals of the edge weights using nonparametric bootstrapping with 1,000 bootstrap samples (28). Larger confidence intervals indicate lower precision of the estimated edge weights. |

**Supplementary Table 5.** Regularized partial correlation matrix of the full cognitive symptom network.

|  | Cog  *ver mem* | Cog  *vis mem* | Cog  *IPS* | Cog  *ver flu* | Cog  *att* | Cog  *inhib* | SCC | HADS  *anx* | HADS  *dep* | CIS  *sub* | CIS  *con* | CIS  *mot* | CIS  *act* |
| --- | --- | --- | --- | --- | --- | --- | --- | --- | --- | --- | --- | --- | --- |
| Cog *ver mem* |  | 0.293 | 0.188 | 0.188 | 0.118 | 0.046 | 0.021 | 0 | 0 | 0 | 0 | 0 | 0 |
| Cog *vis mem* |  |  | 0.211 | 0.004 | 0 | 0.058 | 0 | 0 | 0 | -0.006 | 0 | 0 | 0 |
| Cog *IPS* |  |  |  | 0 | 0.371 | 0.137 | 0 | 0 | 0.020 | 0 | 0 | 0 | 0 |
| Cog *ver flu* |  |  |  |  | 0.143 | 0.057 | -0.041 | -0.063 | 0 | 0 | -0.024 | 0 | 0.077 |
| Cog *att* |  |  |  |  |  | 0.230 | 0.063 | 0 | 0.033 | 0 | 0 | 0 | 0.046 |
| Cog *inhib* |  |  |  |  |  |  | 0.068 | 0.036 | 0.054 | 0 | 0.011 | 0 | 0.038 |
| SCC |  |  |  |  |  |  |  | 0.049 | 0.064 | 0.068 | 0.585 | 0.005 | 0.013 |
| HADS *anx* |  |  |  |  |  |  |  |  | 0.526 | 0.045 | 0.039 | 0 | 0 |
| HADS *dep* |  |  |  |  |  |  |  |  |  | 0.126 | 0.041 | 0.134 | 0.027 |
| CIS *sub* |  |  |  |  |  |  |  |  |  |  | 0.126 | 0.235 | 0.340 |
| CIS *con* |  |  |  |  |  |  |  |  |  |  |  | 0.072 | 0.088 |
| CIS *mot* |  |  |  |  |  |  |  |  |  |  |  |  | 0.264 |
| CIS *act* |  |  |  |  |  |  |  |  |  |  |  |  |  |

This is the regularized partial correlation matrix that is used as input for the visualization of the cognitive symptom network all PwMS (*n* = 703). Each number represents an edge and a zero indicates the absence of an edge. Blue indicates a positive relation and red a negative relation.

*Abbreviations: PwMS = People with MS; HADS = Hospital Anxiety and Depression Scale; SCC = Self-reported Cognitive Complaints; CIS20-R = Checklist Individual Strength (CIS) -20 Revised; Cog = Cognitive function; att = attention; inhib = inhibition; IPS = Information Processing Speed; ver flu = verbal fluency; ver mem = verbal memory; vis mem = visuospatial memory; HADS anx = HADS anxiety subscale; HADS dep = HADS depression subscale; CIS sub = CIS-subjective; CIS con = CIS-concentration; CIS mot = CIS-motivation; CIS act = CIS-activity.*

**
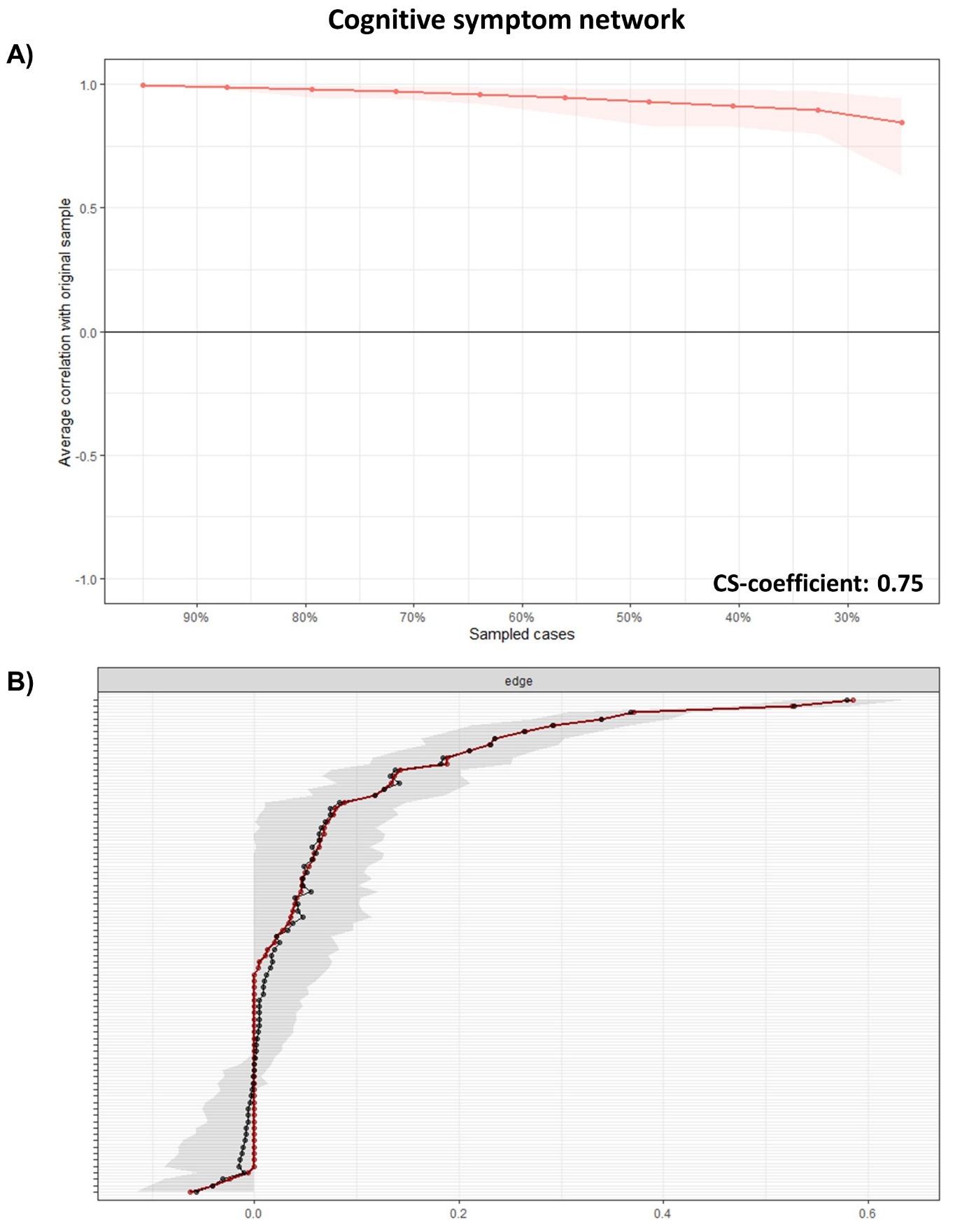
**

**Supplementary Figure 1.** The stability of the nodes and the accuracy of the edge weights for the overall cognitive symptom network (*n* = 703). A) Case-dropping bootstrap, with on the x-axis the percentage of sampled cases dropped with decreases by 10% each time, and on the y-axis the average correlation of the centrality stability coefficient (CS-coefficient) with the original sample. B) Bootstrapped 95% confidence intervals of the edge weights, with on the y-axis all edges in the network ordered from the largest to smallest from top to bottom, and on the x-axis the confidence interval range. The red line represents the edge weights of the network, and the grey bars indicate the 95% confidence intervals around the edge weights.

*Abbreviations: CS-coefficient = centrality stability coefficient.*

**
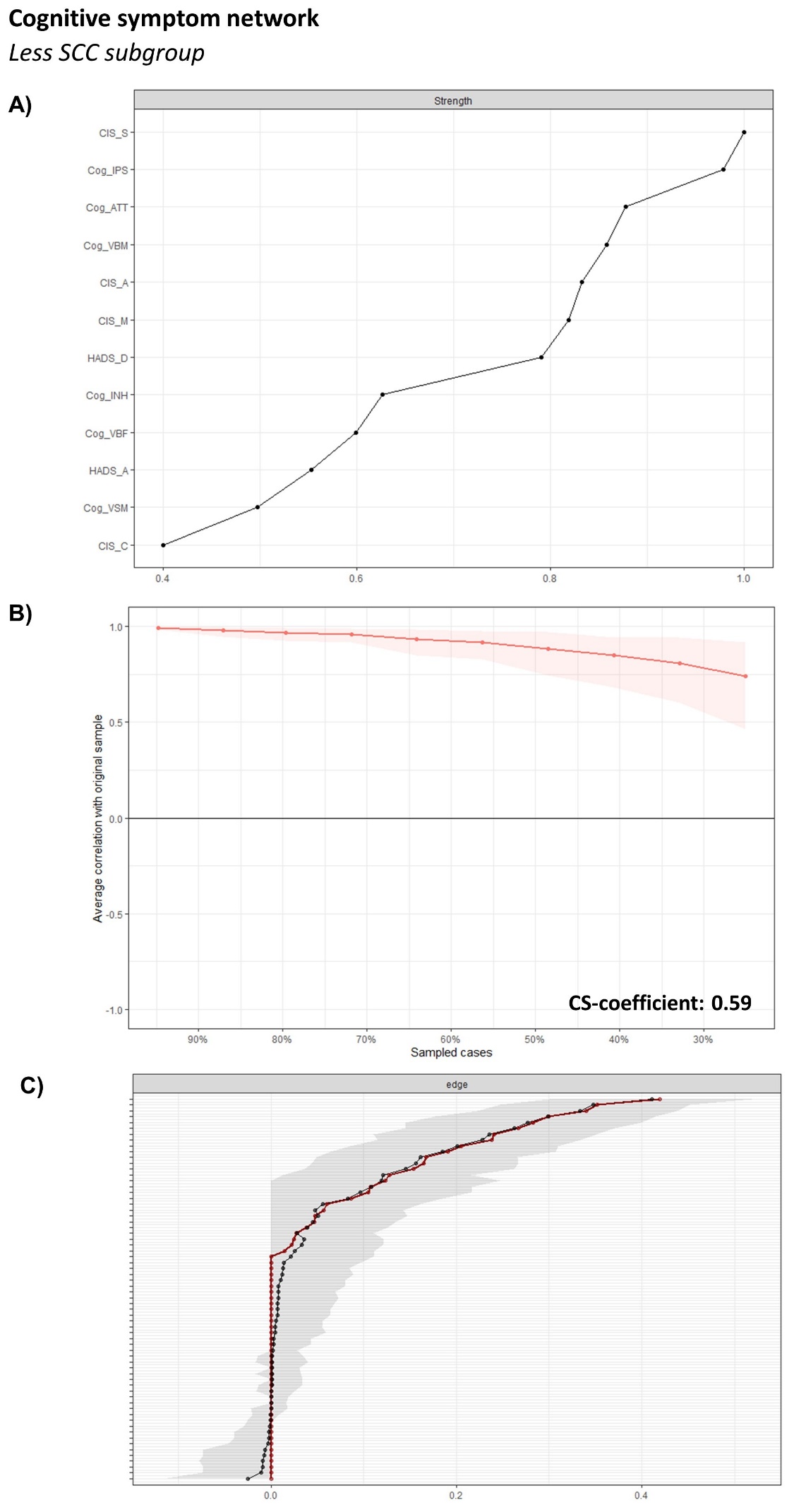
**

**Supplementary Figure 2.** The strength and the stability of the nodes and the accuracy of the edge weights for the cognitive symptom network within the ‘less SCC’ subgroup (*n* = 231). A) Node strength, with on the y-axis all 12 nodes, and the node strength on the x-axis. B) Case-dropping bootstrap, with on the x-axis the percentage of sampled cases dropped with decreases by 10% each time, and on the y-axis the average correlation of the centrality stability coefficient (CS-coefficient) with the original sample. C) Bootstrapped 95% confidence intervals of the edge weights, with on the y-axis all edges in the network ordered from the largest to smallest from top to bottom, and on the x-axis the confidence interval range. The red line represents the edge weights of the network, and the grey bars indicate the 95% confidence intervals around the edge weights.

*Abbreviations: HADS = Hospital Anxiety and Depression Scale; SCC = Self-reported Cognitive Complaints; CIS20-R = Checklist Individual Strength (CIS) -20 Revised; Cog = Cognitive domain; ATT = attention; INH = inhibition; IPS = information processing speed; VBF = verbal fluency; VBM = verbal memory; VSM = visuospatial memory; HADS_A = HADS anxiety subscale; HADS_D = HADS depression subscale; CIS_S = CIS-subjective; CIS_C = CIS-concentration; CIS_M = CIS-motivation; CIS_A = CIS-activity; CS-coefficient = centrality stability coefficient.*

**
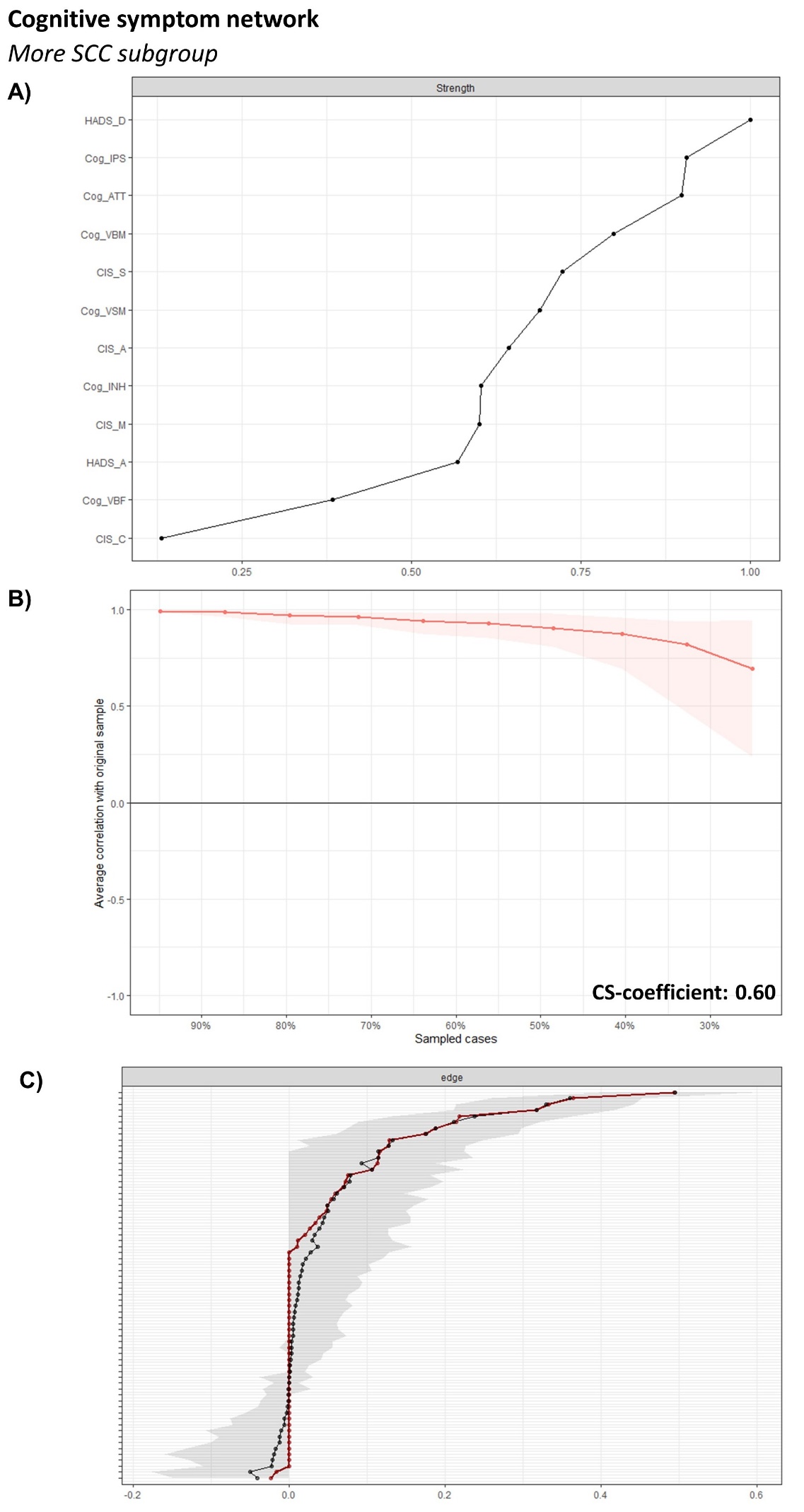
**

**Supplementary Figure 3.** The strength and the stability of the nodes and the accuracy of the edge weights for the cognitive symptom network within the ‘more SCC’ subgroup (*n* = 235). A) Node strength, with on the y-axis all 12 nodes, and the node strength on the x-axis. B) Case-dropping bootstrap, with on the x-axis the percentage of sampled cases dropped with decreases by 10% each time, and on the y-axis the average correlation of the centrality stability coefficient (CS-coefficient) with the original sample. C) Bootstrapped 95% confidence intervals of the edge weights, with on the y-axis all edges in the network ordered from the largest to smallest from top to bottom, and on the x-axis the confidence interval range. The red line represents the edge weights of the network, and the grey bars indicate the 95% confidence intervals around the edge weights.

*Abbreviations: HADS = Hospital Anxiety and Depression Scale; SCC = Self-reported Cognitive Complaints; CIS20-R = Checklist Individual Strength (CIS) -20 Revised; Cog = Cognitive domain; ATT = attention; INH = inhibition; IPS = information processing speed; VBF = verbal fluency; VBM = verbal memory; VSM = visuospatial memory; HADS_A = HADS anxiety subscale; HADS_D = HADS depression subscale; CIS_S = CIS-subjective; CIS_C = CIS-concentration; CIS_M = CIS-motivation; CIS_A = CIS-activity; CS-coefficient = centrality stability coefficient.*

**
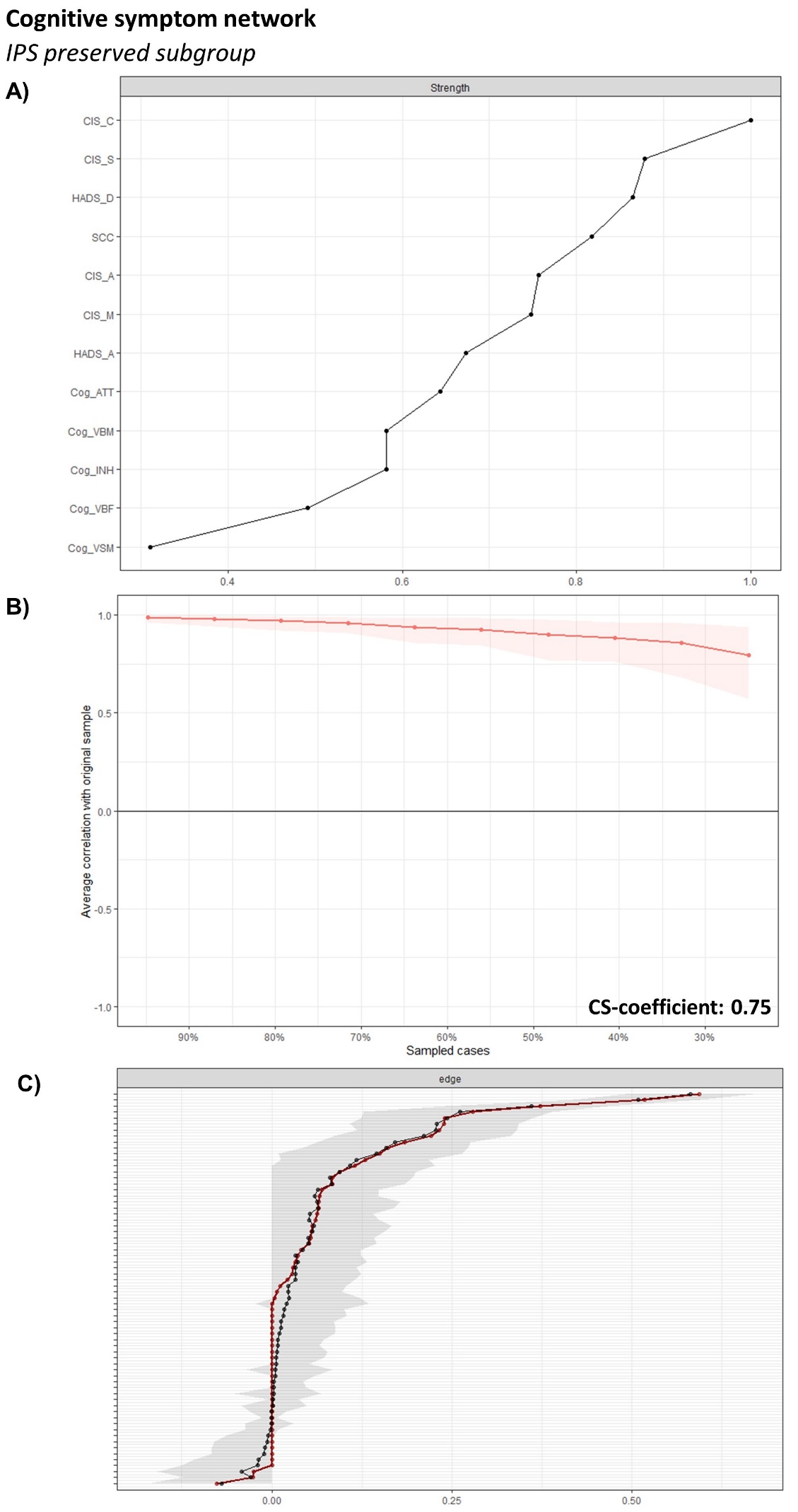
**

**Supplementary Figure 4.** The strength and the stability of the nodes and the accuracy of the edge weights for the cognitive symptom network within the ‘IPS preserved’ subgroup (*n* = 463). A) Node strength, with on the y-axis all 12 nodes, and the node strength on the x-axis. B) Case-dropping bootstrap, with on the x-axis the percentage of sampled cases dropped with decreases by 10% each time, and on the y-axis the average correlation of the centrality stability coefficient (CS-coefficient) with the original sample. C) Bootstrapped 95% confidence intervals of the edge weights, with on the y-axis all edges in the network ordered from the largest to smallest from top to bottom, and on the x-axis the confidence interval range. The red line represents the edge weights of the network, and the grey bars indicate the 95% confidence intervals around the edge weights.

*Abbreviations: HADS = Hospital Anxiety and Depression Scale; SCC = Self-reported Cognitive Complaints; CIS20-R = Checklist Individual Strength (CIS) -20 Revised; Cog = Cognitive domain; ATT = attention; INH = inhibition; IPS = information processing speed; VBF = verbal fluency; VBM = verbal memory; VSM = visuospatial memory; HADS_A = HADS anxiety subscale; HADS_D = HADS depression subscale; CIS_S = CIS-subjective; CIS_C = CIS-concentration; CIS_M = CIS-motivation; CIS_A = CIS-activity; CS-coefficient = centrality stability coefficient.*

**
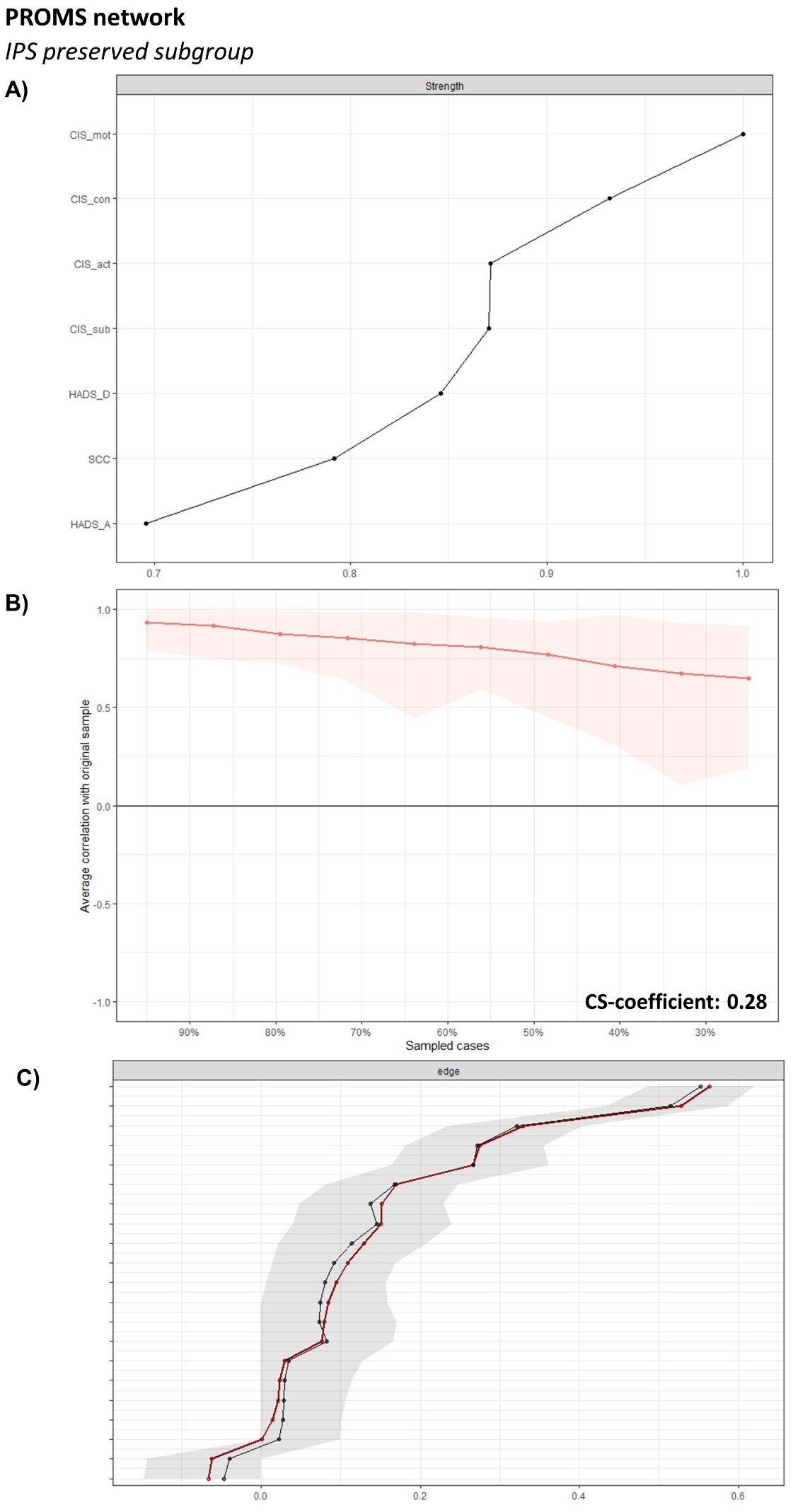
**

**Supplementary Figure 5.** The strength and the stability of the nodes and the accuracy of the edge weights for the PROMS network within the ‘IPS preserved’ subgroup (*n* = 463). A) Node strength, with on the y-axis all 7 nodes, and the node strength on the x-axis. B) Case-dropping bootstrap, with on the x-axis the percentage of sampled cases dropped with decreases by 10% each time, and on the y-axis the average correlation of the centrality stability coefficient (CS-coefficient) with the original sample. C) Bootstrapped 95% confidence intervals of the edge weights, with on the y-axis all edges in the network ordered from the largest to smallest from top to bottom, and on the x-axis the confidence interval range. The red line represents the edge weights of the network, and the grey bars indicate the 95% confidence intervals around the edge weights.

*Abbreviations: HADS = Hospital Anxiety and Depression Scale; SCC = Self-reported Cognitive Complaints; CIS20-R = Checklist Individual Strength (CIS) -20 Revised; IPS = information processing speed; HADS_A = HADS anxiety subscale; HADS_D = HADS depression subscale; CIS_S = CIS-subjective; CIS_C = CIS-concentration; CIS_M = CIS-motivation; CIS_A = CIS-activity; CS-coefficient = centrality stability coefficient.*


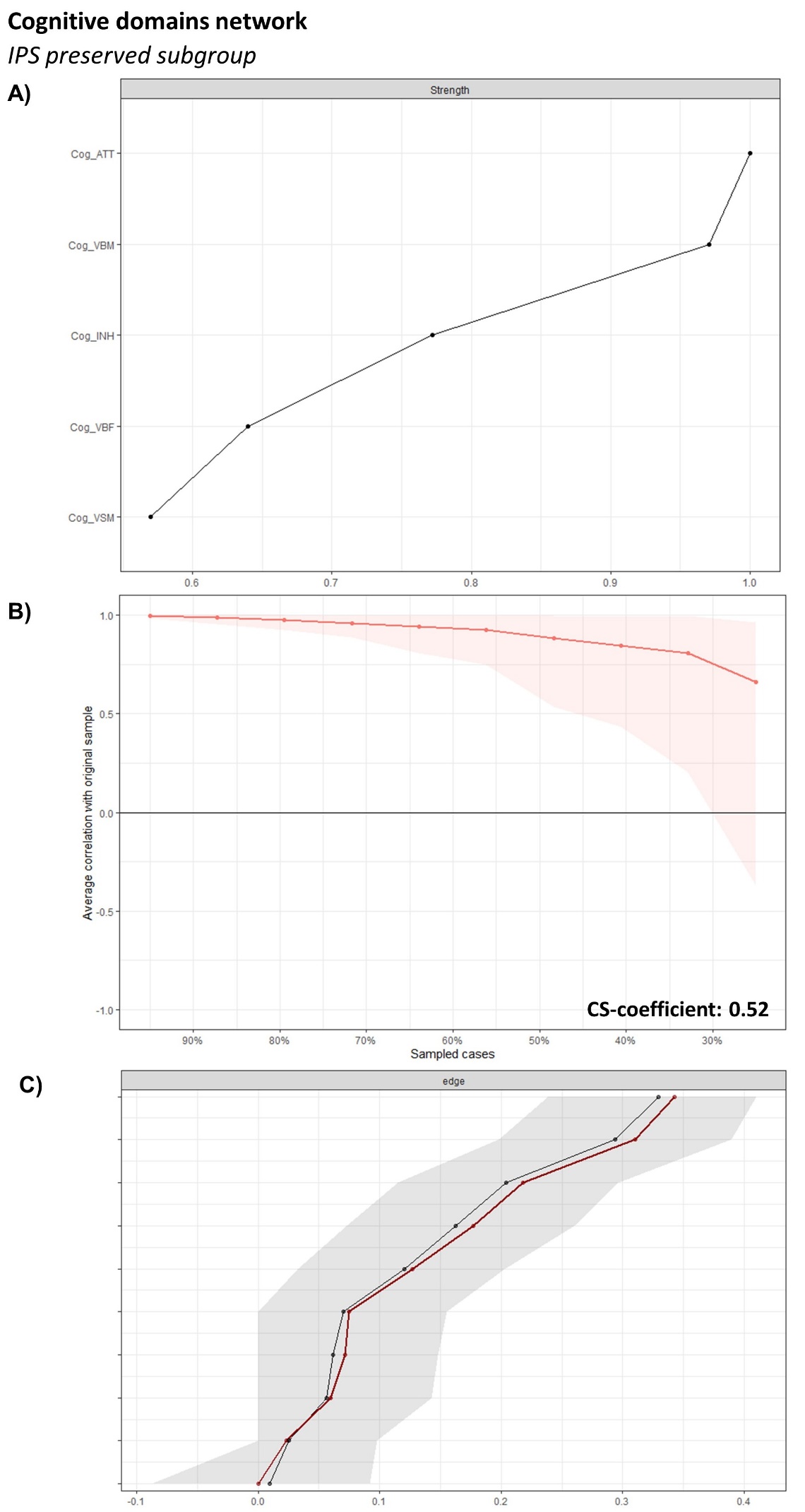


**Supplementary Figure 6.** The strength and the stability of the nodes and the accuracy of the edge weights for the cognitive domains network within the ‘IPS preserved’ subgroup (*n* = 463). A) Node strength, with on the y-axis all 5 nodes, and the node strength on the x-axis. B) Case-dropping bootstrap, with on the x-axis the percentage of sampled cases dropped with decreases by 10% each time, and on the y-axis the average correlation of the centrality stability coefficient (CS-coefficient) with the original sample. C) Bootstrapped 95% confidence intervals of the edge weights, with on the y-axis all edges in the network ordered from the largest to smallest from top to bottom, and on the x-axis the confidence interval range. The red line represents the edge weights of the network, and the grey bars indicate the 95% confidence intervals around the edge weights.

*Abbreviations: Cog = Cognitive domain; ATT = attention; INH = inhibition; IPS = information processing speed; VBF = verbal fluency; VBM = verbal memory; VSM = visuospatial memory; CS-coefficient = centrality stability coefficient.*

**
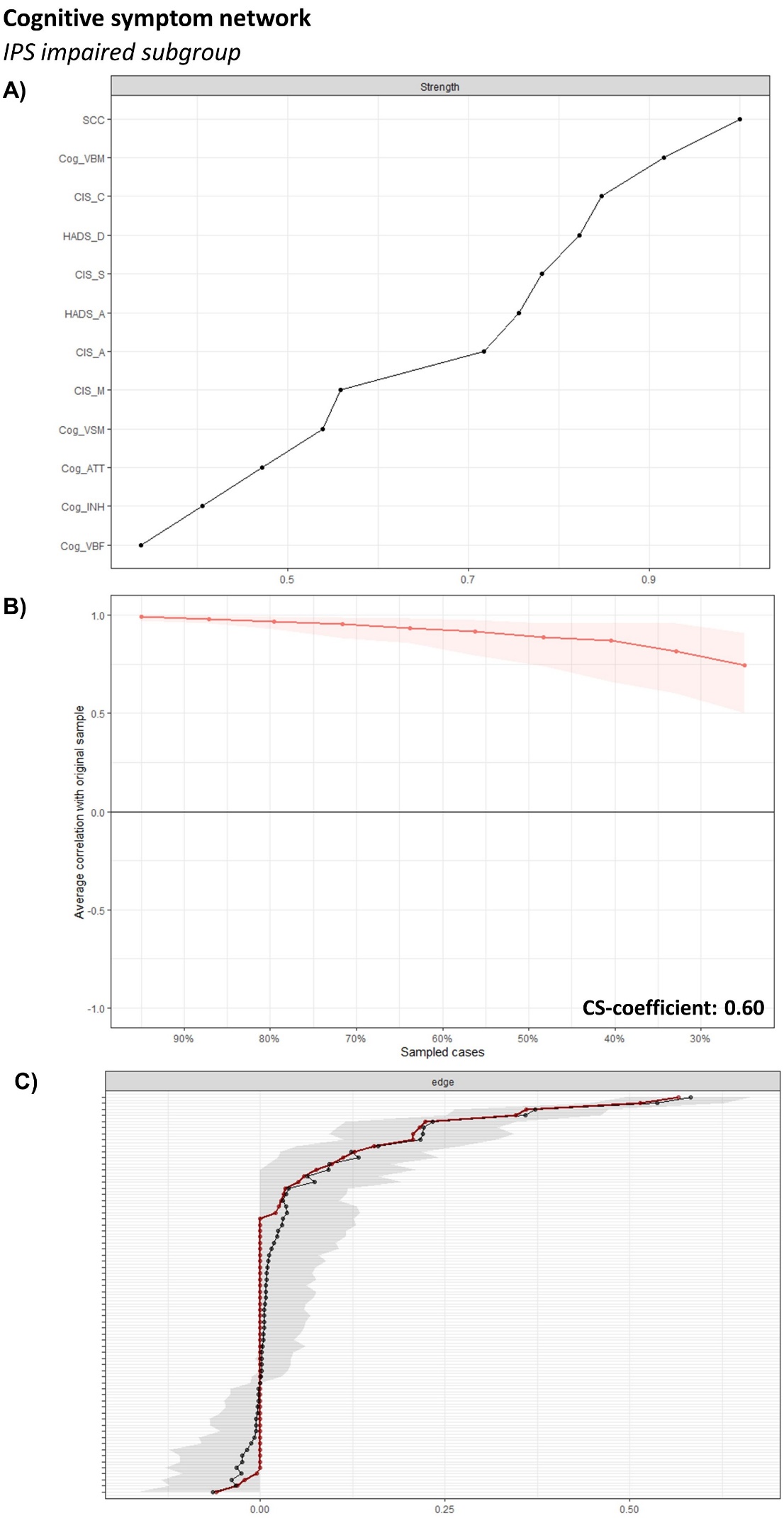
**

**Supplementary Figure 7.** The strength and the stability of the nodes and the accuracy of the edge weights for the cognitive symptom network within the ‘IPS impaired’ subgroup (*n* = 240). A) Node strength, with on the y-axis all 12 nodes, and the node strength on the x-axis. B) Case-dropping bootstrap, with on the x-axis the percentage of sampled cases dropped with decreases by 10% each time, and on the y-axis the average correlation of the centrality stability coefficient (CS-coefficient) with the original sample. C) Bootstrapped 95% confidence intervals of the edge weights, with on the y-axis all edges in the network ordered from the largest to smallest from top to bottom, and on the x-axis the confidence interval range. The red line represents the edge weights of the network, and the grey bars indicate the 95% confidence intervals around the edge weights.

*Abbreviations: HADS = Hospital Anxiety and Depression Scale; SCC = Self-reported Cognitive Complaints; CIS20-R = Checklist Individual Strength (CIS) -20 Revised; Cog = Cognitive domain; ATT = attention; INH = inhibition; IPS = information processing speed; VBF = verbal fluency; VBM = verbal memory; VSM = visuospatial memory; HADS_A = HADS anxiety subscale; HADS_D = HADS depression subscale; CIS_S = CIS-subjective; CIS_C = CIS-concentration; CIS_M = CIS-motivation; CIS_A = CIS-activity; CS-coefficient = centrality stability coefficient.*

**
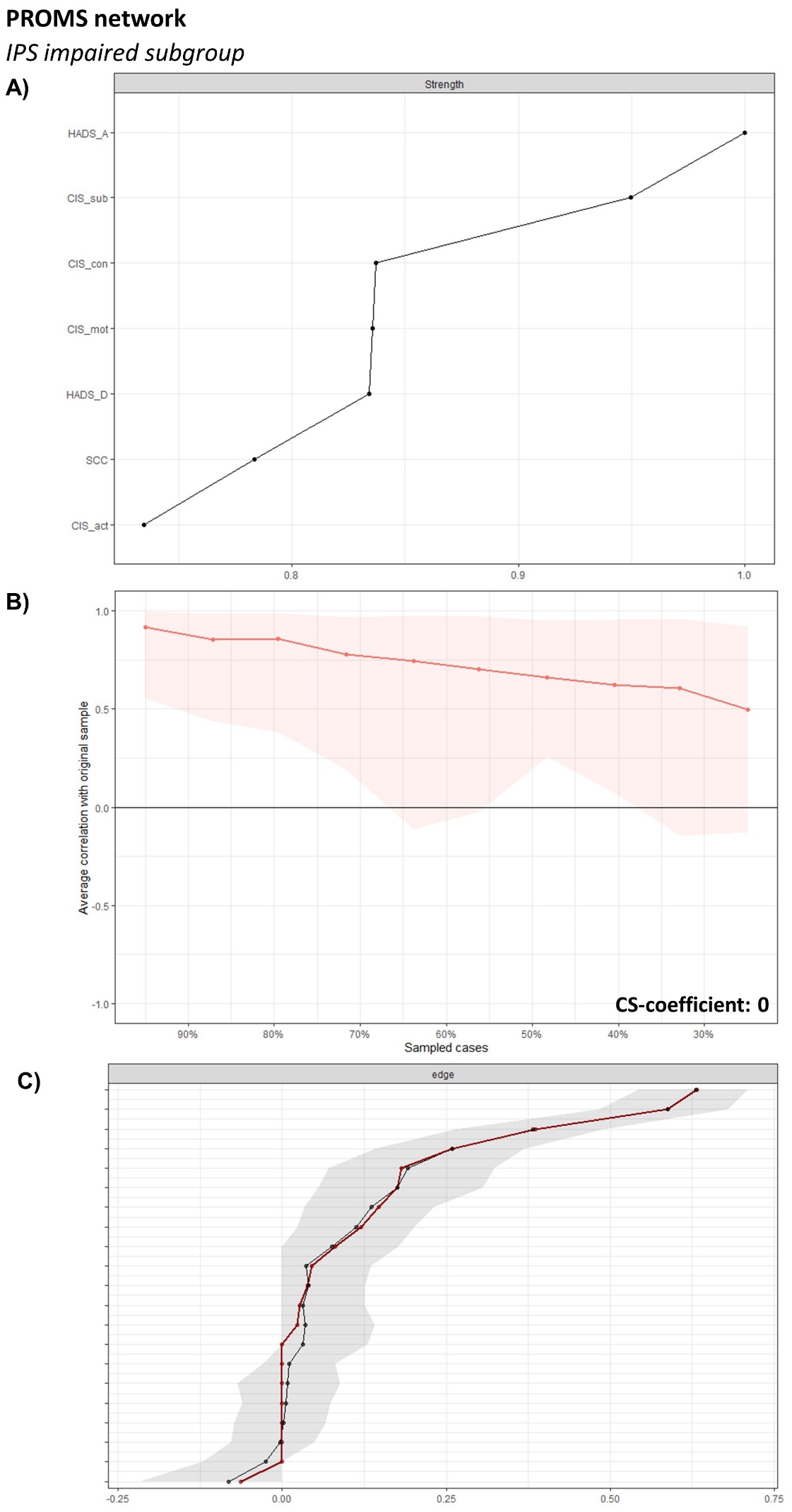
**

**Supplementary Figure 8.** The strength and the stability of the nodes and the accuracy of the edge weights for the PROMS network within the ‘IPS impaired’ subgroup (*n* = 240). A) Node strength, with on the y-axis all 7 nodes, and the node strength on the x-axis. B) Case-dropping bootstrap, with on the x-axis the percentage of sampled cases dropped with decreases by 10% each time, and on the y-axis the average correlation of the centrality stability coefficient (CS-coefficient) with the original sample. C) Bootstrapped 95% confidence intervals of the edge weights, with on the y-axis all edges in the network ordered from the largest to smallest from top to bottom, and on the x-axis the confidence interval range. The red line represents the edge weights of the network, and the grey bars indicate the 95% confidence intervals around the edge weights.

*Abbreviations: HADS = Hospital Anxiety and Depression Scale; SCC = Self-reported Cognitive Complaints; CIS20-R = Checklist Individual Strength (CIS) -20 Revised; IPS = information processing speed; HADS_D = HADS depression subscale; CIS_S = CIS-subjective; CIS_C = CIS-concentration; CIS_M = CIS-motivation; CIS_A = CIS-activity; CS-coefficient = centrality stability coefficient.*

**
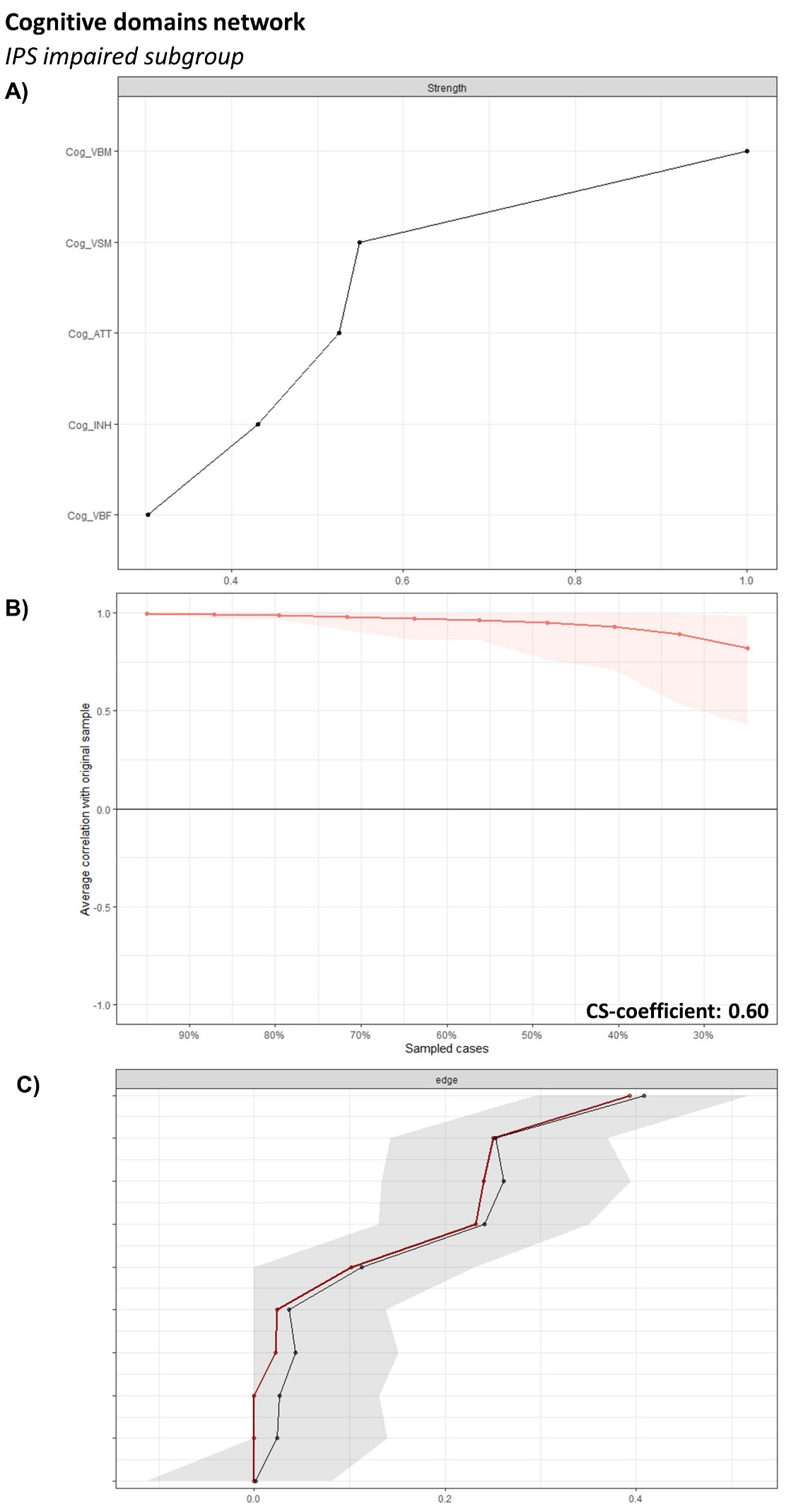
**

**Supplementary Figure 9.** The strength and the stability of the nodes and the accuracy of the edge weights for the cognitive domains network within the ‘IPS impaired’ subgroup (*n* = 240). A) Node strength, with on the y-axis all 5 nodes, and the node strength on the x-axis. B) Case-dropping bootstrap, with on the x-axis the percentage of sampled cases dropped with decreases by 10% each time, and on the y-axis the average correlation of the centrality stability coefficient (CS-coefficient) with the original sample. C) Bootstrapped 95% confidence intervals of the edge weights, with on the y-axis all edges in the network ordered from the largest to smallest from top to bottom, and on the x-axis the confidence interval range. The red line represents the edge weights of the network, and the grey bars indicate the 95% confidence intervals around the edge weights.

*Abbreviations: Cog = Cognitive domain; ATT = attention; INH = inhibition; IPS = information processing speed; VBF = verbal fluency; VBM = verbal memory; VSM = visuospatial memory; CS-coefficient = centrality stability coefficient.*

**Appendix A – split based on IPS functioning in PwMS**

Importantly, not all PwMS in the ‘higher IPS tertile’ subgroup (indicating poorer performance) had an IPS impairment based on normative data (*n* = 45, 18.8%, χ^2^ = 388.94, *p* <.001). In the ‘higher IPS tertile’ subgroup (indicating worse performance), 40 PwMS (17.1%) exhibited no IPS impairment. The networks based on ‘lower and higher IPS tertiles’ are included in Supplementary Figure 10. Supplementary Figures 11 and 12 summarize the strength and stability of the nodes in these networks, as well as the accuracy of the edges. The global strength was not significantly different between the ‘lower IPS tertile’ and ‘better IPS tertile’ networks (global strength = 4.64 versus 3.86, respectively, *p* = .080).


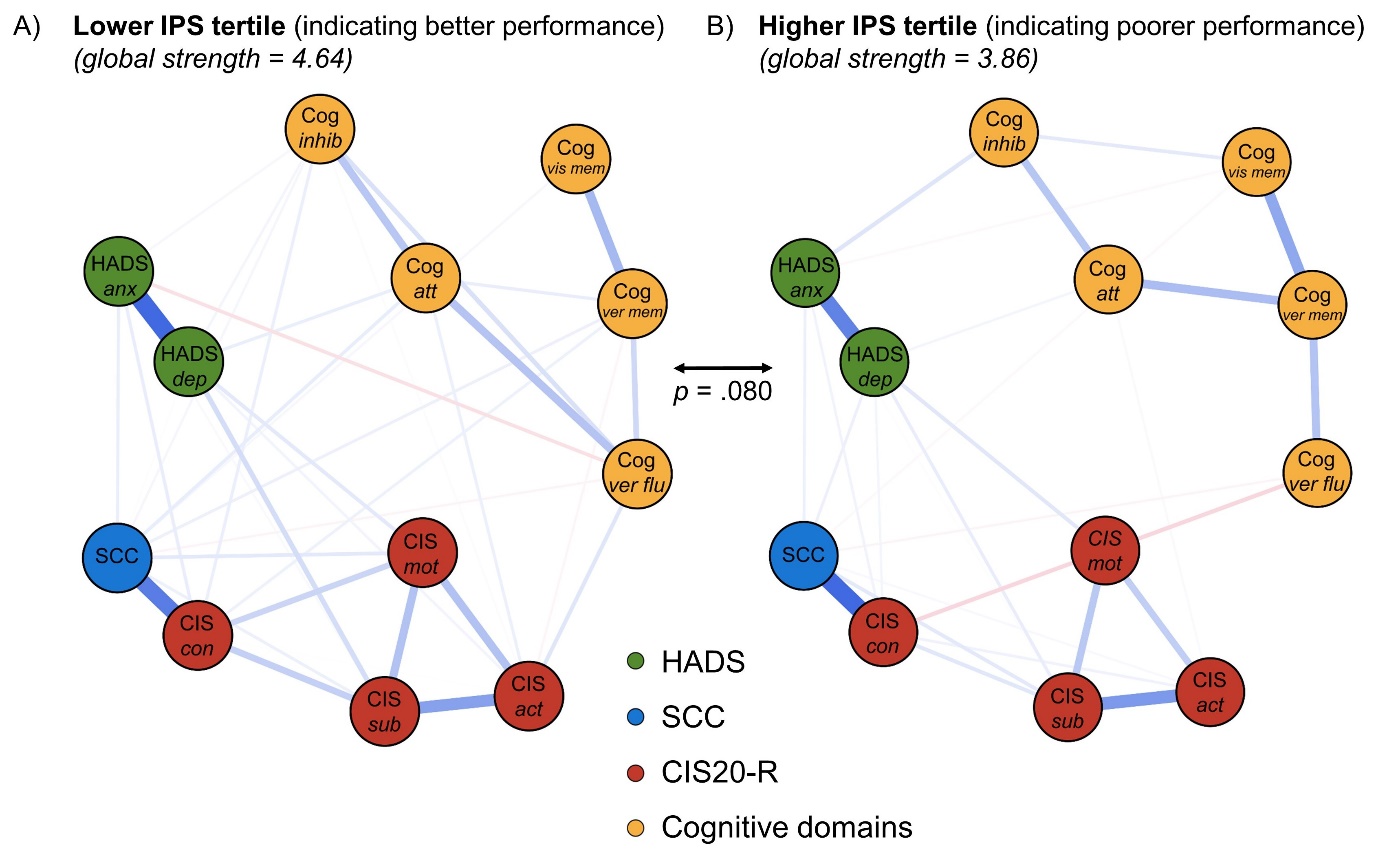


**Supplementary Figure 10.** The cognitive symptom networks in PwMS – split based on IPS functioning in PwMS. A) The cognitive symptom network in PwMS in the ‘lower IPS tertile’ (indicating better performance on IPS, *n* = 232). B) The cognitive symptom network in PwMS in the ‘higher IPS tertile’ (indicating poorer performance on IPS, *n* = 234). The colors of the nodes refer to the corresponding PROMS or cognitive domains. A blue edge indicates a positive relationship between the two nodes and a red edge a negative relationship. Edges were undirected and weighted and in the presented figures, edge width corresponds to the magnitude of the association.

*Abbreviations: PwMS = People with MS; HADS = Hospital Anxiety and Depression Scale; SCC = Self-reported Cognitive Complaints; CIS20-R = Checklist Individual Strength (CIS)-20 Revised; Cog = Cognitive domain; att = attention; inhib = inhibition; IPS = information processing speed; ver flu = verbal fluency; ver mem = verbal memory; vis mem = visuospatial memory; HADS anx = HADS anxiety subscale; HADS dep = HADS depression subscale; CIS sub = CIS-subjective; CIS con = CIS-concentration; CIS mot = CIS-motivation; CIS act = CIS-activity.*


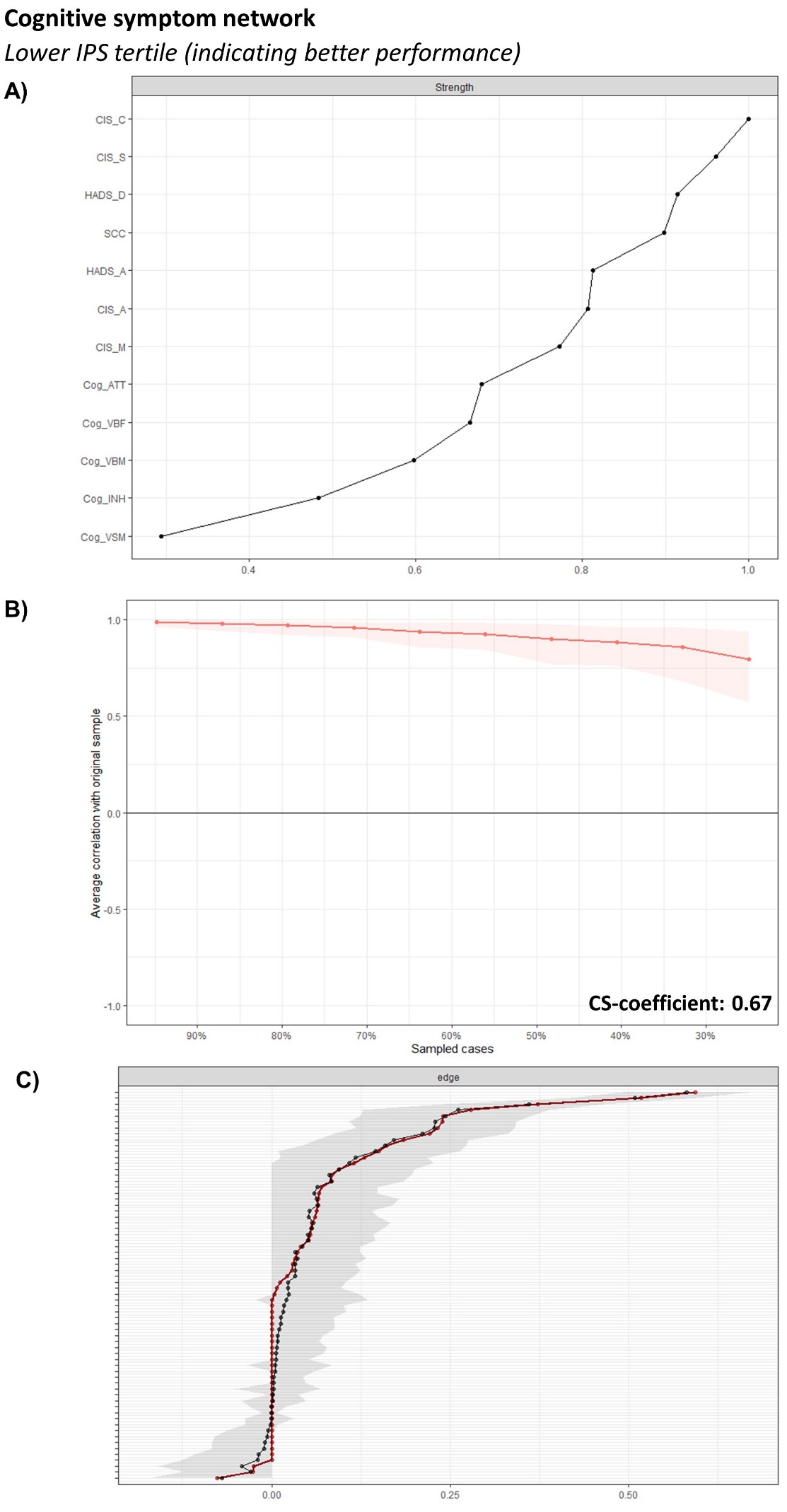


**Supplementary Figure 11.** The strength and the stability of the nodes and the accuracy of the edge weights for the cognitive symptom network within the ‘lower IPS tertile’ subgroup (indicating better performance, *n* = 232). A) Node strength, with on the y-axis all 12 nodes, and the node strength on the x-axis. B) Case-dropping bootstrap, with on the x-axis the percentage of sampled cases dropped with decreases by 10% each time, and on the y-axis the average correlation of the centrality stability coefficient (CS-coefficient) with the original sample. C) Bootstrapped 95% confidence intervals of the edge weights, with on the y-axis all edges in the network ordered from the largest to smallest from top to bottom, and on the x-axis the confidence interval range. The red line represents the edge weights of the network, and the grey bars indicate the 95% confidence intervals around the edge weights.

*Abbreviations: HADS = Hospital Anxiety and Depression Scale; SCC = Self-reported Cognitive Complaints; CIS20-R = Checklist Individual Strength (CIS) -20 Revised; Cog = Cognitive domain; ATT = attention; INH = inhibition; IPS = information processing speed; VBF = verbal fluency; VBM = verbal memory; VSM = visuospatial memory; HADS_A = HADS anxiety subscale; HADS_D = HADS depression subscale; CIS_S = CIS-subjective; CIS_C = CIS-concentration; CIS_M = CIS-motivation; CIS_A = CIS-activity; CS-coefficient = centrality stability coefficient.*

**
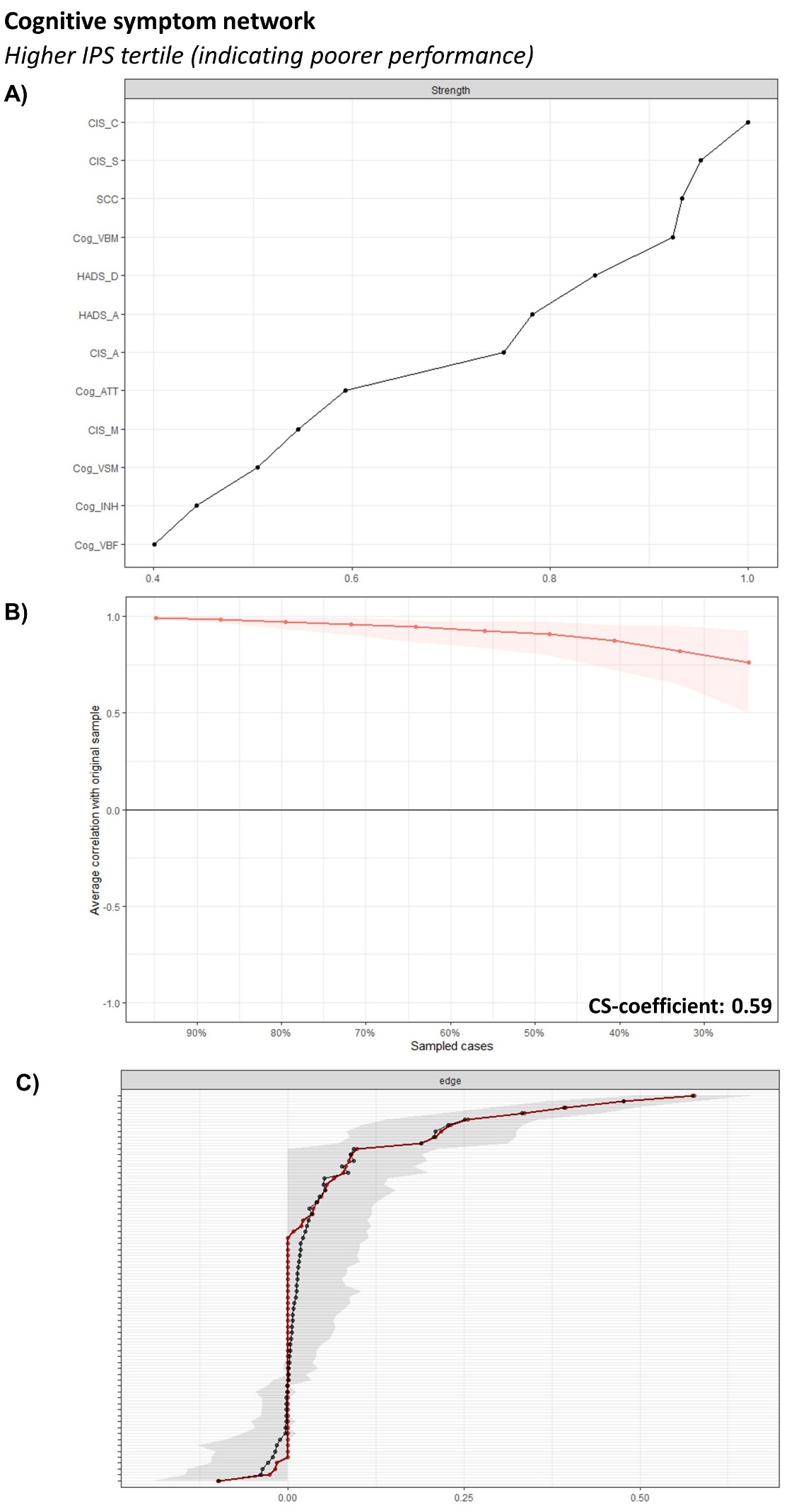
**

**Supplementary Figure 12.** The strength and the stability of the nodes and the accuracy of the edge weights for the cognitive symptom network within the ‘higher IPS tertile’ subgroup (indicating poorer performance, *n* = 234). A) Node strength, with on the y-axis all 12 nodes, and the node strength on the x-axis. B) Case-dropping bootstrap, with on the x-axis the percentage of sampled cases dropped with decreases by 10% each time, and on the y-axis the average correlation of the centrality stability coefficient (CS-coefficient) with the original sample. C) Bootstrapped 95% confidence intervals of the edge weights, with on the y-axis all edges in the network ordered from the largest to smallest from top to bottom, and on the x-axis the confidence interval range. The red line represents the edge weights of the network, and the grey bars indicate the 95% confidence intervals around the edge weights.

*Abbreviations: HADS = Hospital Anxiety and Depression Scale; SCC = Self-reported Cognitive Complaints; CIS20-R = Checklist Individual Strength (CIS) -20 Revised; Cog = Cognitive domain; ATT = attention; INH = inhibition; IPS = information processing speed; VBF = verbal fluency; VBM = verbal memory; VSM = visuospatial memory; HADS_A = HADS anxiety subscale; HADS_D = HADS depression subscale; CIS_S = CIS-subjective; CIS_C = CIS-concentration; CIS_M = CIS-motivation; CIS_A = CIS-activity; CS-coefficient = centrality stability coefficient.*

**Appendix B – split based on sex**

The networks based on sex are in included in Supplementary Figure 13. Supplementary Figures 14 and 15 summarize the strength and stability of the nodes in these networks, as well as the accuracy of the edges. The global strength was not significantly different between the females and males networks (global strength = 5.55 versus 5.22, respectively, *p* = .470).

**
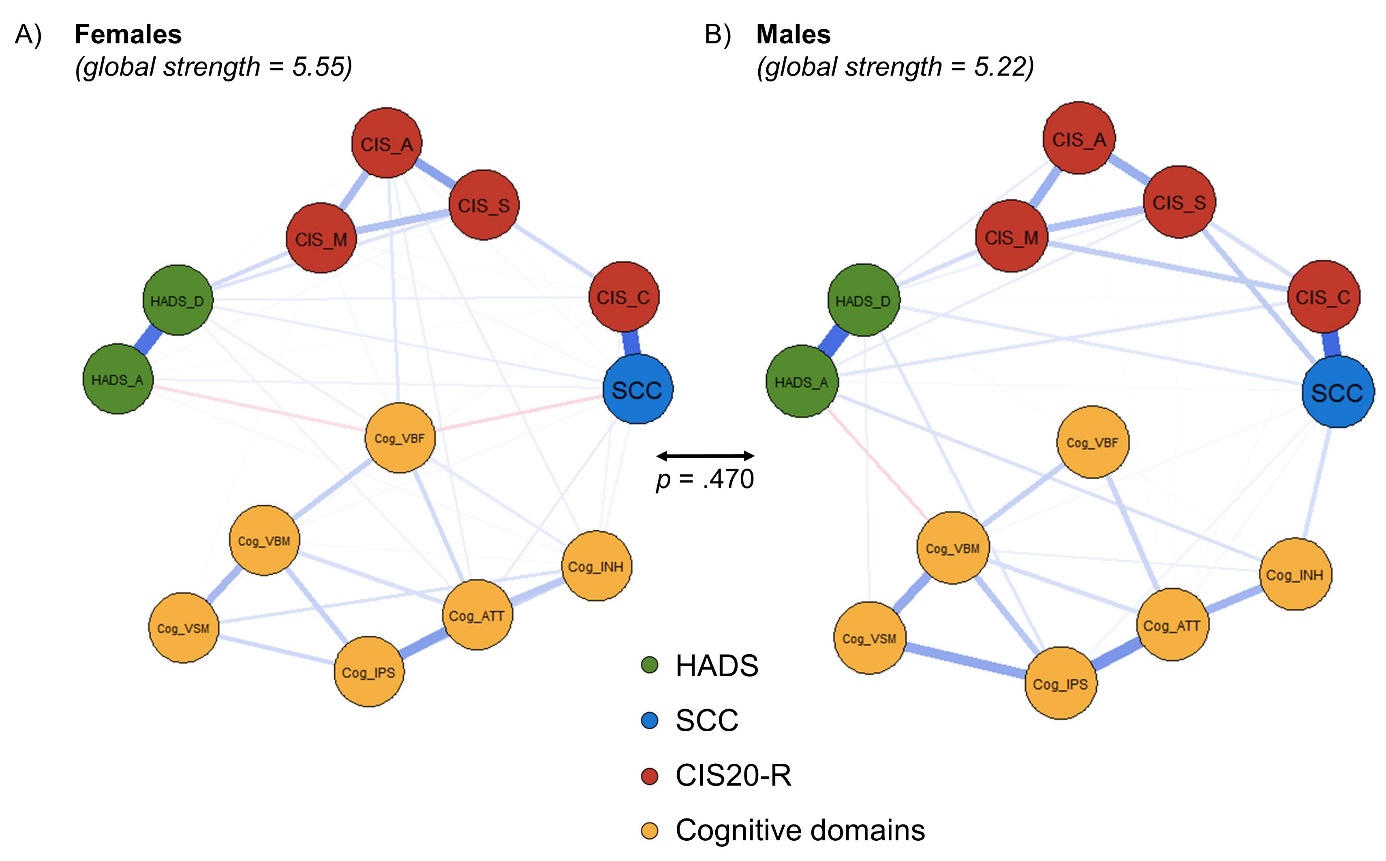
**

**Supplementary Figure 13.** The cognitive symptom networks in PwMS – split based on sex. A) The cognitive symptom network in females (*n* = 501). B) The cognitive symptom network in males (*n* = 202). The colors of the nodes refer to the corresponding PROMS or cognitive domains. A blue edge indicates a positive relationship between the two nodes and a red edge a negative relationship. Edges were undirected and weighted and in the presented figures, edge width corresponds to the magnitude of the association.

*Abbreviations: HADS = Hospital Anxiety and Depression Scale; SCC = Self-reported Cognitive Complaints; CIS20-R = Checklist Individual Strength (CIS) -20 Revised; Cog = Cognitive domain; ATT = attention; INH = inhibition; IPS = information processing speed; VBF = verbal fluency; VBM = verbal memory; VSM = visuospatial memory; HADS_A = HADS anxiety subscale; HADS_D = HADS depression subscale; CIS_S = CIS-subjective; CIS_C = CIS-concentration; CIS_M = CIS-motivation; CIS_A = CIS-activity; CS-coefficient = centrality stability coefficient.*

**
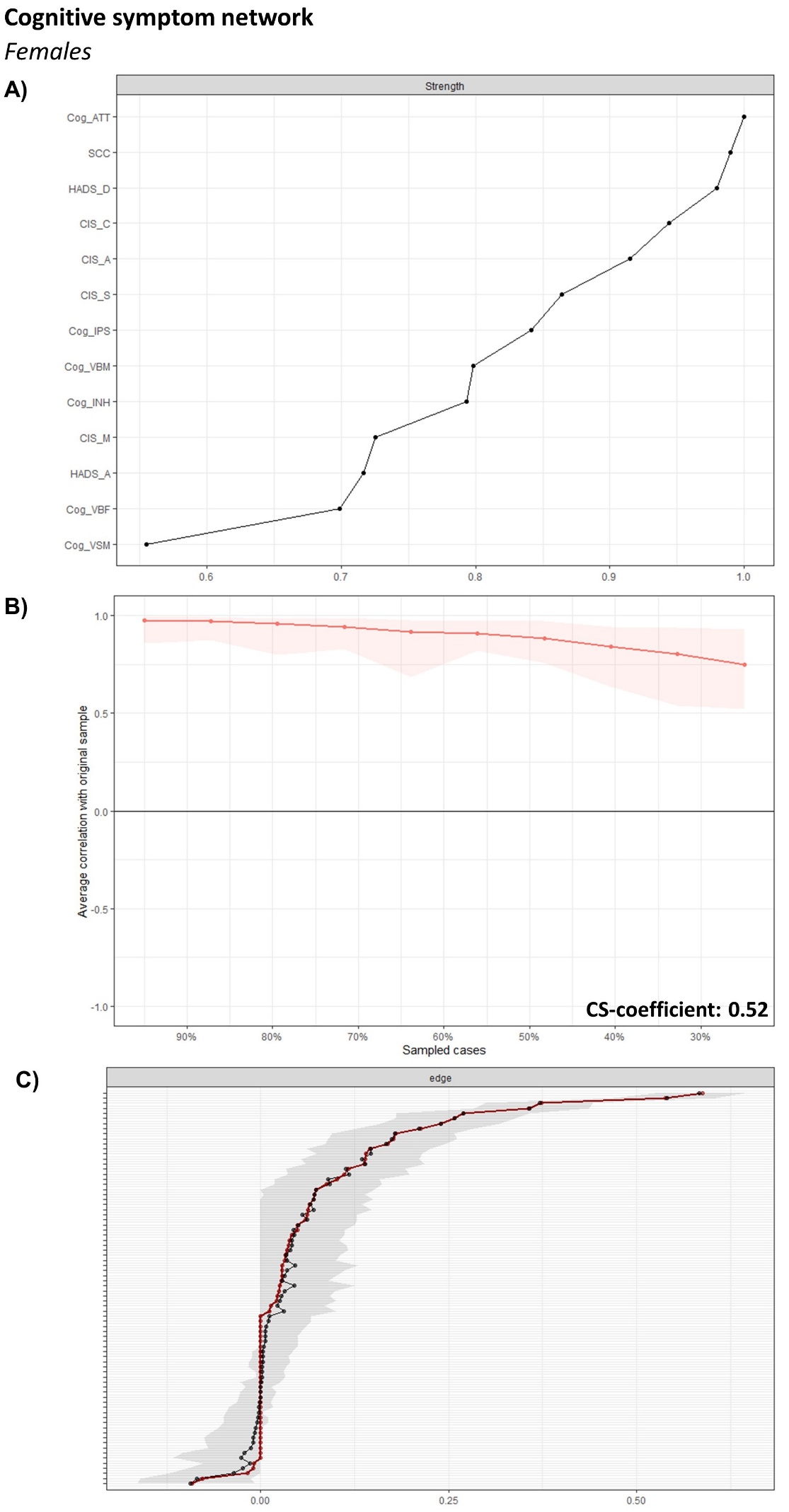
**

**Supplementary Figure 14.** The strength and the stability of the nodes and the accuracy of the edge weights for the cognitive symptom network within females (*n* = 501). A) Node strength, with on the y-axis all 13 nodes, and the node strength on the x-axis. B) Case-dropping bootstrap, with on the x-axis the percentage of sampled cases dropped with decreases by 10% each time, and on the y-axis the average correlation of the centrality stability coefficient (CS-coefficient) with the original sample. C) Bootstrapped 95% confidence intervals of the edge weights, with on the y-axis all edges in the network ordered from the largest to smallest from top to bottom, and on the x-axis the confidence interval range. The red line represents the edge weights of the network, and the grey bars indicate the 95% confidence intervals around the edge weights.

*Abbreviations: HADS = Hospital Anxiety and Depression Scale; SCC = Self-reported Cognitive Complaints; CIS20-R = Checklist Individual Strength (CIS) -20 Revised; Cog = Cognitive domain; ATT = attention; INH = inhibition; IPS = information processing speed; VBF = verbal fluency; VBM = verbal memory; VSM = visuospatial memory; HADS_A = HADS anxiety subscale; HADS_D = HADS depression subscale; CIS_S = CIS-subjective; CIS_C = CIS-concentration; CIS_M = CIS-motivation; CIS_A = CIS-activity; CS-coefficient = centrality stability coefficient.*

**
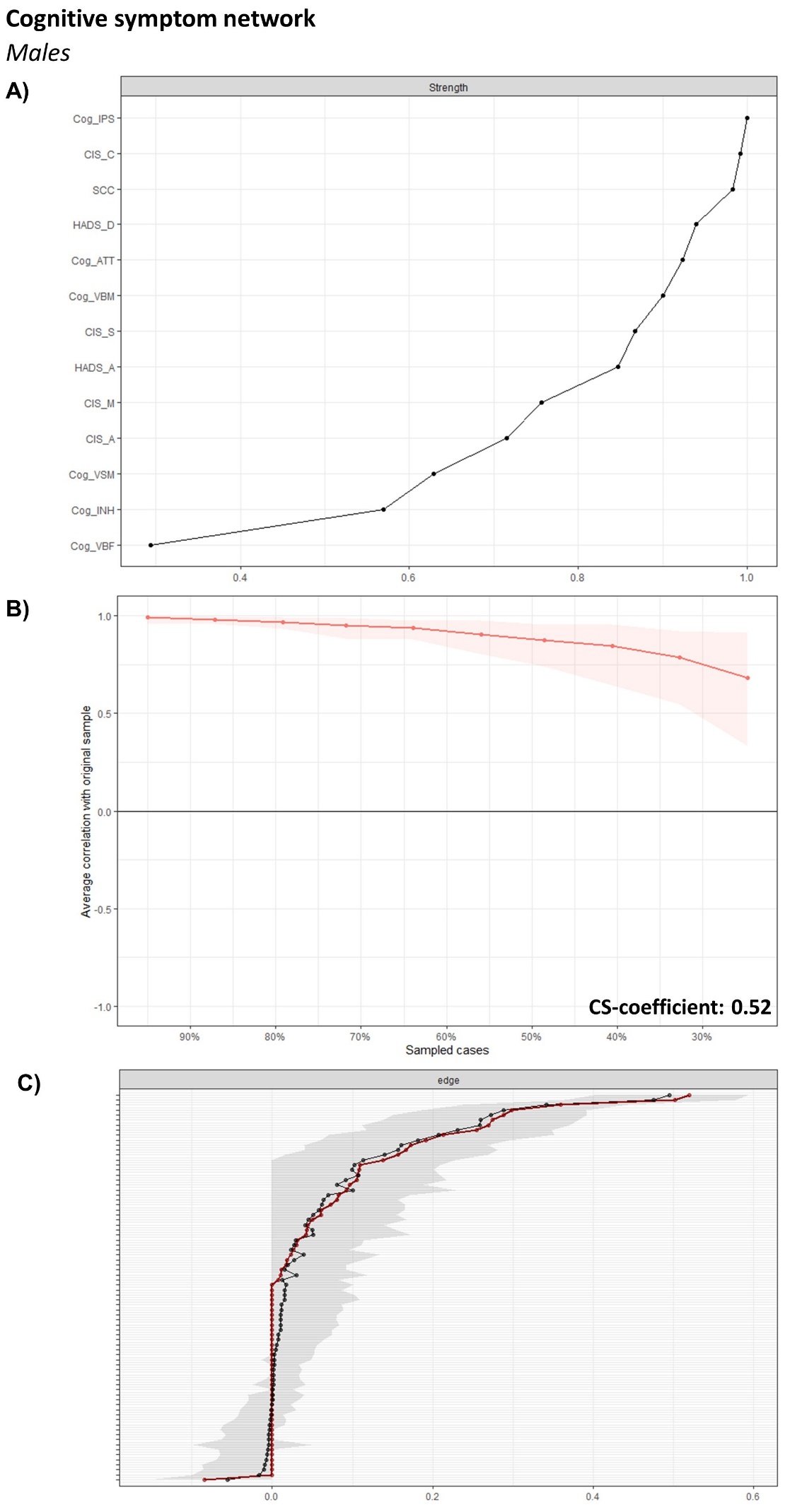
**

**Supplementary Figure 15.** The strength and the stability of the nodes and the accuracy of the edge weights for the cognitive symptom network within males (*n* = 202). A) Node strength, with on the y-axis all 13 nodes, and the node strength on the x-axis. B) Case-dropping bootstrap, with on the x-axis the percentage of sampled cases dropped with decreases by 10% each time, and on the y-axis the average correlation of the centrality stability coefficient (CS-coefficient) with the original sample. C) Bootstrapped 95% confidence intervals of the edge weights, with on the y-axis all edges in the network ordered from the largest to smallest from top to bottom, and on the x-axis the confidence interval range. The red line represents the edge weights of the network, and the grey bars indicate the 95% confidence intervals around the edge weights.

*Abbreviations: HADS = Hospital Anxiety and Depression Scale; SCC = Self-reported Cognitive Complaints; CIS20-R = Checklist Individual Strength (CIS) -20 Revised; Cog = Cognitive domain; ATT = attention; INH = inhibition; IPS = information processing speed; VBF = verbal fluency; VBM = verbal memory; VSM = visuospatial memory; HADS_A = HADS anxiety subscale; HADS_D = HADS depression subscale; CIS_S = CIS-subjective; CIS_C = CIS-concentration; CIS_M = CIS-motivation; CIS_A = CIS-activity; CS-coefficient = centrality stability coefficient.*

**Supplemental references**

1. Prouskas SE, Schoonheim MM, Huiskamp M, Steenwijk MD, Gehring K, Barkhof F, et al. A randomized trial predicting response to cognitive rehabilitation in multiple sclerosis: Is there a window of opportunity? Multiple Sclerosis Journal. 2022;28(13):2124-36.

2. Polman CH, Reingold SC, Banwell B, Clanet M, Cohen JA, Filippi M, et al. Diagnostic criteria for multiple sclerosis: 2010 revisions to the McDonald criteria. Annals of neurology. 2011;69(2):292-302.

3. Schoonheim MM, Vigeveno RM, Lopes FCR, Pouwels PJ, Polman CH, Barkhof F, Geurts JJ. Sex‐specific extent and severity of white matter damage in multiple sclerosis: Implications for cognitive decline. Human brain mapping. 2014;35(5):2348-58.

4. Steenwijk MD, Daams M, Pouwels PJ, Balk LJ, Tewarie PK, Killestein J, et al. What explains gray matter atrophy in long-standing multiple sclerosis? Radiology. 2014;272(3):832-42.

5. Schoonheim MM, Hulst HE, Brandt RB, Strik M, Wink AM, Uitdehaag BM, et al. Thalamus structure and function determine severity of cognitive impairment in multiple sclerosis. Neurology. 2015;84(8):776-83.

6. Steenwijk MD, Daams M, Pouwels PJ, J. Balk L, Tewarie PK, Geurts JJ, et al. Unraveling the relationship between regional gray matter atrophy and pathology in connected white matter tracts in long‐standing multiple sclerosis. Human brain mapping. 2015;36(5):1796-807.

7. Daams M, Steenwijk MD, Schoonheim MM, Wattjes MP, Balk LJ, Tewarie PK, et al. Multi-parametric structural magnetic resonance imaging in relation to cognitive dysfunction in long-standing multiple sclerosis. Multiple Sclerosis Journal. 2016;22(5):608-19.

8. Eijlers AJ, Meijer KA, Wassenaar TM, Steenwijk MD, Uitdehaag BM, Barkhof F, et al. Increased default-mode network centrality in cognitively impaired multiple sclerosis patients. Neurology. 2017;88(10):952-60.

9. Meijer KA, Eijlers AJ, Douw L, Uitdehaag BM, Barkhof F, Geurts JJ, Schoonheim MM. Increased connectivity of hub networks and cognitive impairment in multiple sclerosis. Neurology. 2017;88(22):2107-14.

10. Eijlers AJ, Meijer KA, van Geest Q, Geurts JJ, Schoonheim MM. Determinants of cognitive impairment in patients with multiple sclerosis with and without atrophy. Radiology. 2018;288(2):544-51.

11. Eijlers AJ, van Geest Q, Dekker I, Steenwijk MD, Meijer KA, Hulst HE, et al. Predicting cognitive decline in multiple sclerosis: a 5-year follow-up study. Brain. 2018;141(9):2605-18.

12. Meijer K, Van Geest Q, Eijlers A, Geurts J, Schoonheim M, Hulst H. Is impaired information processing speed a matter of structural or functional damage in MS? NeuroImage: Clinical. 2018;20:844-50.

13. Eijlers AJ, Wink AM, Meijer KA, Douw L, Geurts JJ, Schoonheim MM. Reduced network dynamics on functional MRI signals cognitive impairment in multiple sclerosis. Radiology. 2019;292(2):449-57.

14. Nauta IM, Balk LJ, Sonder JM, Hulst HE, Uitdehaag BM, Fasotti L, de Jong BA. The clinical value of the patient-reported multiple sclerosis neuropsychological screening questionnaire. Multiple Sclerosis Journal. 2019;25(11):1543-6.

15. Huiskamp M, Eijlers AJ, Broeders TA, Pasteuning J, Dekker I, Uitdehaag BM, et al. Longitudinal network changes and conversion to cognitive impairment in multiple sclerosis. Neurology. 2021;97(8):e794-e802.

16. Schoonheim MM, Pinter D, Prouskas SE, Broeders TA, Pirpamer L, Khalil M, et al. Disability in multiple sclerosis is related to thalamic connectivity and cortical network atrophy. Multiple Sclerosis Journal. 2021:13524585211008743.

17. Schoonheim MM, Douw L, Broeders TA, Eijlers AJ, Meijer KA, Geurts JJ. The cerebellum and its network: Disrupted static and dynamic functional connectivity patterns and cognitive impairment in multiple sclerosis. Multiple Sclerosis Journal. 2021;27(13):2031-9.

18. Strik M, Chard DT, Dekker I, Meijer KA, Eijlers AJ, Pardini M, et al. Increased functional sensorimotor network efficiency relates to disability in multiple sclerosis. Multiple Sclerosis Journal. 2021;27(9):1364-73.

19. Broeders TA, Douw L, Eijlers AJ, Dekker I, Uitdehaag BM, Barkhof F, et al. A more unstable resting-state functional network in cognitively declining multiple sclerosis. Brain Communications. 2022;4(2):fcac095.

20. Kulik SD, Nauta IM, Tewarie P, Koubiyr I, van Dellen E, Ruet A, et al. Structure-function coupling as a correlate and potential biomarker of cognitive impairment in multiple sclerosis. Network Neuroscience. 2022;6(2):339-56.

21. Tijhuis FB, Broeders TAA, Santos FAN, Schoonheim MM, Killestein J, Leurs CE, et al. Dynamic functional connectivity as a neural correlate of fatigue in multiple sclerosis. NeuroImage: Clinical. 2021;29:102556.

22. Huiskamp M, Yaqub M, van Lingen MR, Pouwels PJ, de Ruiter LR, Killestein J, et al. Cognitive performance in multiple sclerosis: what is the role of the gamma-aminobutyric acid system? Brain Communications. 2023;5(3):fcad140.

23. Thompson AJ, Banwell BL, Barkhof F, Carroll WM, Coetzee T, Comi G, et al. Diagnosis of multiple sclerosis: 2017 revisions of the McDonald criteria. The Lancet Neurology. 2018;17(2):162-73.

24. Nauta IM, Bertens D, Fasotti L, Fieldhouse J, Uitdehaag BM, Kessels RP, et al. Cognitive rehabilitation and mindfulness reduce cognitive complaints in multiple sclerosis (REMIND-MS): a randomized controlled trial. Multiple Sclerosis and Related Disorders. 2023:104529.

25. Nauta IM, Speckens AE, Kessels RP, Geurts JJ, de Groot V, Uitdehaag BM, et al. Cognitive rehabilitation and mindfulness in multiple sclerosis (REMIND-MS): a study protocol for a randomised controlled trial. BMC neurology. 2017;17(1):1-10.

26. van Dam M, de Jong BA, Willemse EA, Nauta IM, Huiskamp M, Klein M, et al. A multimodal marker for cognitive functioning in multiple sclerosis: the role of NfL, GFAP and conventional MRI in predicting cognitive functioning in a prospective clinical cohort. Journal of Neurology. 2023:1-11.

27. Nauta I, Bertens D, van Dam M, Huiskamp M, Driessen S, Geurts J, et al. Performance validity in outpatients with multiple sclerosis and cognitive complaints. Multiple Sclerosis Journal.0(0):13524585211025780.

28. Epskamp S, Borsboom D, Fried EI. Estimating psychological networks and their accuracy: A tutorial paper. Behavior Research Methods. 2018;50(1):195-212.
